# Neurokinin-1 receptor activation protects against cardiac fibrosis, inflammation and diastolic dysfunction in type 2 diabetic mice

**DOI:** 10.1111/bph.70440

**Published:** 2026-04-25

**Authors:** Alexander Widiapradja, Heather Connery, Brody J. Helmick, Kallie J. Schafner, Vamsi Manchikalapudi, Hannah Horvath, Sophia Gugino, Tamas Kriska, Anja Herrnreiter, Evan DeVallance, William B. Campbell, Scott P. Levick

**Affiliations:** 1 Robert C. Byrd Health Sciences Center, Department of Physiology, Pharmacology and Toxicology, West Virginia University, Morgantown, West Virginia, USA; 2 Department of Pharmacology and Toxicology, Medical College of Wisconsin, Milwaukee, Wisconsin, USA

**Keywords:** cardiac fibrosis, diastolic function, fibroblast, heart failure, macrophage, substance P

## Abstract

**Background and Purpose:**

The pathogenesis of type 2 diabetes mellitus (T2DM)-induced cardiomyopathy involves cardiac fibrosis that leads to diastolic dysfunction. We established that replacement of lost substance P (SP) that occurs in T2DM reduces cardiac fibrosis and decreases inflammation in T2DM mice and non-human primates. This study aimed to identify the specific anti-fibrotic SP receptor.

**Experimental Approach:**

Age-matched male wild type (WT) and Lepr^db/db^ mice at 12 weeks of age were treated with either saline or the neurokinin-1 receptor (NK-1R) agonist, GR73632 (300 μg·kg^−1^·day^−1^) for 4 weeks. The left ventricles were assessed for cardiac function, fibrosis, mast cells and macrophage phenotype. Mouse cardiac fibroblast and bone marrow-derived macrophage cultures were exposed to high glucose and treated with GR73632 to assess collagen release, signalling pathways and cytokine release respectively. Proteomics analysis was conducted to assess the left ventricular proteomic profile between WT and Lepr^db/db^ mice, and the effects of GR73632.

**Key Results:**

NK-1R activation decreased cardiac fibrosis, improved diastolic function, decreased mast cell numbers and promoted an anti-inflammatory macrophage phenotype in Lepr^db/db^ mice. NK-1R activation reduced collagen I production by high glucose treated mouse cardiac fibroblasts. NK-1R activation decreased P65 phosphorylation (NF-*κ*B) and CCL2 chemokine release. Proteomic analysis revealed a distinct proteome profile between WT and Lepr^db/db^ mouse hearts.

**Conclusion and Implications:**

The NK-1R is the anti-fibrotic SP receptor and improves diastolic function in the diabetic heart. This likely involves direct effects on cardiac fibroblasts and macrophages. This study provides a potential target for treatment of diabetic cardiomyopathy.

## 1 | INTRODUCTION

Type 2 diabetes mellitus (T2DM) patients have a 20%–40% higher incidence of heart failure, along with a 33% greater risk of hospitalisation (Borghetti et al., 2018; Russo & Frangogiannis, 2016). The pathogenesis of T2DM-induced heart failure is often associated with hypertension and coronary artery disease. However, a specific diabetic cardiomyopathy including cardiomyocyte hypertrophy, micro-vascular damage and interstitial fibrosis can occur independent of other comorbidities (Hölscher et al., 2016; Marwick et al., 2018; Wong et al., 2014). Cardiac fibrosis leads to impaired left ventricular (LV) compliance and diastolic dysfunction that ultimately manifests as heart failure with preserved ejection fraction (HFpEF). Despite the increased morbidity and mortality associated with T2DM-induced cardiac fibrosis, there are no effective treatment strategies to ameliorate this fibrosis and diastolic dysfunction characteristic of HFpEF (González et al., 2018). One of the consequences of T2DM is peripheral neuropathy that results in the loss of neuropeptides. The loss of specific neuropeptides can predispose the heart to developing diabetic cardiomyopathy. We have established that, in serum, there is a loss of the neuropeptide substance P (SP) in T2DM Lepr^db/db^ mice, concomitant with cardiac fibrosis; replacement SP significantly reduced cardiac fibrosis. In addition, replacement SP also reduced inflammation and promoted an anti-inflammatory M2 macrophage phenotype (Widiapradja et al., 2021). We demonstrated the translational relevance of replacement SP in a Vervet monkey pre-clinical model of T2DM that also had reduced plasma SP (Meléndez et al., 2023). Replacement SP in these monkeys resulted in reduced fibrosis and improved cardiac function.

The pressing question following these replacement SP studies was, which receptor mediates the antifibrotic actions of SP? The cognate receptor for SP is the neurokinin-1 receptor (NK-1R) (Steinhoff et al., 2014). However, SP also activates the neurokinin-2 receptor (NK-2R) and neurokinin-3 receptor (NK-3R), albeit at lower affinities (Regoli et al., 1988). We chose to focus on the NK-1R in this study. To examine whether the NK-1R is the anti-fibrotic SP receptor, we utilised GR73632, a selective NK-1R agonist (Brocco et al., 2008; Rupniak et al., 2000; Rupniak et al., 2001), in T2DM Lepr^db/db^ mice.

## 2 | METHODS

### 2.1 | Experimental design

Age matched 12-week-old male wild type (WT) C57BL/6 mice and Lepr^db/db^ (B6.BKS(D)-Lepr^*db*^/J) mice were purchased from Jackson Laboratories and housed under standard environmental conditions with commercial mouse chow and tap water ad libitum. WT and Lepr^db/db^ mice were then assigned to the following experimental groups: (1) WT (n = 16); (2) Lepr^db/db^ + saline (n = 23); (3) Lepr^db/db^ + GR73632 (300-μg·kg^−1^·day^−1^ subcutaneous injection, n = 23); and (4) Lepr^db/db^ + L732138 (5-mg·kg^−1^·day^−1^ intraperitoneal injection, n = 5). GR73632, is a highly selective NK-1R agonist, and L732138 is a selective NK-1R antagonist. Treatments were administered daily for 4 weeks, thereby, at the end of the treatment period, all mice were 16 weeks of age. The dose of GR73632 was determined by a pilot study that found no detrimental side effects, whilst reducing cardiac fibrosis and improving cardiac function. The dose of L732138 was determined in our previous studies (Dehlin et al., 2013; Widiapradja et al., 2019). A separate group of 12-week-old, male WT (n = 8) and Lepr^db/db^ (n = 8) mice receiving no treatment were included to determine whether specific parameters were reversed or prevented by NK-1R activation. Cardiac function was assessed in anaesthetised mice (2% inhaled isoflurane) by pressure-volume catheterisation. Palpebral reflex, toe pinch reflex and corneal reflex were evaluated for proper analgesia prior to tracheal intubation. A pressure–volume catheter (ADInstrument) was inserted into the LV through the apical portion of the heart, and functional read outs were recorded and analysed using LabChart (ADInstrument). Euthanasia was then accomplished by removal of the heart. The right ventricle (RV) was separated from the LV and septum and weighed. The apical portion of the LV was snap-frozen in liquid nitrogen and stored in −80°C for molecular analysis, and the base was fixed in 10% zinc formalin solution for histological analysis. Lungs and kidneys also were weighed and collected. Tibial length was measured for organ weight normalisation. A set of in-house male 8-week-old LacZ^+^/Tacr1^fl/fl^ NK-1R reporter mice were euthanised and the hearts were collected and fixed in 10% zinc formalin solution for histological analysis. Animal studies are reported in compliance with the ARRIVE guidelines (Percie du Sert et al., 2020) and with the recommendations made by the British Journal of Pharmacology (Lilley et al., 2020). These studies conformed to the principles of the National Health Institute of Health Guide for the Care and Use of Laboratory Animals, and the protocol was approved by the Institutional Animal Care and Use Committee (IACUC) under the approval reference number 2208056770.1 and 2203051915_R1.

### 2.2 | Collagen volume fraction

Cardiac fibrosis was assessed using collagen volume fraction as determined from picrosirius red staining for fibrillar collagen. Formalin fixed LV sections of 5 μm thickness were deparaffinised and rehydrated before incubation in 0.2% of phosphomolybdic acid solution and staining with picrosirius red (0.1% Sirius Red F3BA in picric acid). The slides were dehydrated before being mounted and cover slipped with DEPEX. The slides were then scanned using a VS120AW Olympus Slide Scanner at 200X magnification and the collagen volume fraction was analysed using Fiji Image J software (National Institute of Health).

### 2.3 | Wall-to-lumen ratio

Wall-to-lumen ratio (WLR) for LV intra-cardiac arteries was determined using Olympus VS200 software from images obtained from a VS120AW Olympus Slide Scanner at 200X magnification. The area of the entire vessel (VA) ending at the media-adventitia border line, and the area of the arterial lumen (LA) were calculated using the freehand polygon tool. The WLR was calculated using the following equation: WLR = (VA − LA)/LA. Arteries were divided into four groups based on size (<2000, 2000–5000, 5000–10000 and >10000 μm^2^).

### 2.4 | Intracellular calcium mobilisation assay

An intracellular calcium [Ca^2+^]_*i*_ mobilisation assay was performed using a fluorescence imaging plate reader (FLIPR, Molecular Devices) as previously described (Kriska et al., 2022; Park et al., 2018). In brief, human ventricular cardiac fibroblasts (Lonza, Cat# CC-2904) were thawed and directly plated onto a gelatin-coated black-walled clear-bottomed 96-well plate at 6000 cells per well. The cells were grown to confluence over 5 days. The next day, media was removed, and cells were first incubated with Fluo-4 NW (no-wash) dye containing probenecid (2.5 mM) at 37°C for 30 min, followed by another 30-min incubation at room temperature whilst wrapped in foil. Then, the NK-1R antagonist, aprepitant (10 μM), or vehicle was added 20 min into the room temperature incubation. Basal fluorescence was monitored for 20 s, then either 25-μl of SP or GR73632 dilutions were added, and the signal recorded for 200 s. Relative changes in [Ca^2+^]_*i*_ were calculated by subtracting the average basal fluorescence (average fluorescence from 0 to 20 s) from the maximum fluorescence (peak fluorescence value) as previously described (Park et al., 2018). Results were expressed as change in relative fluorescence units (ΔRFU). Experiments were performed on three separate frozen cell stocks.

### 2.5 | Isolation and treatment of cardiac fibroblasts

Mouse cardiac fibroblasts were isolated as described in our previous studies (McCaffrey et al., 2020; Widiapradja et al., 2021; Widiapradja et al., 2024). Briefly, the LV from 8-week-old male WT and Tacr1^−/−^ mice was collected, minced and digested in a series of five incubations in 100 ng·ml^−1^ of Liberase TM (Roche) at 37°C for 15 min. The cells were then seeded and cultured in DMEM-F12 media containing 5-mM glucose with 10% foetal bovine serum (FBS; Life Technologies). Non adherent cells were removed by washing with Moscona’s solution. Once the cultures reached confluency, the fibroblasts were seeded on 0.1% gelatin-coated plates for subsequent treatments. Fibroblasts were passaged only once to minimise phenotype changes associated with passaging. Prior to treatment, fibroblasts were serum starved in DMEM-F12 media for 24 h on 0.1% gelatin-coated plates, then treated with either normal glucose (NG, 5 mM) or high glucose (HG, 25 mM), with or without GR73632 (100 and 300 nM) for 24 h. All treatments were performed in DMEM-F12 media containing 1.5% FBS. Culture media and cells were collected separately and processed for either RNA or protein. WT and *Tacr1*^−*/*−^ cardiac fibroblasts were cultured on sterile coverslips and did not undergo any treatment beyond serum starvation. These cells were fixed in 4%PFA for immunolabelling.

### 2.6 | Isolation and treatment of bone marrow–derived macrophages

Mouse bone marrow-derived macrophages (BMM*θ*) were generated as described in our previous studies (Widiapradja et al., 2021; Widiapradja et al., 2024). Briefly, bone marrow cells were extracted from the tibia and femur of 8-week-old male WT mice and cultured in DMEM supplemented with 1% penicillin/streptomycin and 10% FBS, as well as 10 ng·ml^−1^ of macrophage colony stimulating factor (MCSF) for 10 days. Once confluent, the cells were treated with media containing either NG (5 nM), HG (25 nM) or HG with GR73632 (300 nM) for 6 h. The media were then collected to analyse cytokine release.

### 2.7 | ELISA

Commercially available enzyme-linked immunosorbent assay (ELISA) kits were used to measure collagen Iα1 (Novus Biologicals) in both LV and in culture media from the isolated cardiac fibroblasts. CCL2 (BD Biosciences) was measured in the culture media from isolated cardiac fibroblasts. TNF-α, IL-6 and CCL2 (BD Biosciences) were measured in the culture media from BMM*θ*.

### 2.8 | Protein extraction and immunoblotting

Protein from tissues and cells was extracted in T-Per and RIPA buffer, respectively, containing proteinase and phosphatase inhibitors (Thermo Scientific). Protein in the lysates was then quantified using a Pierce bicinchoninic acid assay (BCA) protein assay kit (Thermo Scientific) according to the manufacturer’s instructions. 20–30 μg of protein from each sample was loaded into 10% polyacrylamide gels for electrophoresis and run to optimal separation before blotting into nitrocellulose membranes. The membranes were then blocked in Intercept (TBS) blocking buffer (Li-Cor) for 1 h at room temperature before undergoing overnight incubation with primary antibodies: rabbit anti mouse p-AKT (1:1000, Cell Signaling Technology Cat# 4058, RRID:AB_331168), rabbit anti mouse Smad7 (1:1000, Abcam Cat# ab272928, RRID:AB_2924382), rabbit anti mouse p-P65 (1:1000, Cell Signaling Technology Cat# 3033, RRID:AB_331284) and rabbit anti mouse GAPDH (1:1000, Sigma-Aldrich Cat# G9545, RRID:AB_796208). Once secondary antibodies (1:10000, goat anti rabbit 700 or 800, Li-Cor) were applied for 1 h, the membranes were imaged using a Li-Cor Odyssey CL-X imager (Li-Cor) with the appropriate laser channel. The proteins of interest were then analysed using Li-Cor Image Studio analysis software and normalised to GAPDH as a house-keeping protein. The experimental details provided conforms with the *British Journal of Pharmacology* Guidelines (Alexander et al., 2018).

### 2.9 | Immunolabelling

The 5-μm formalin fixed LV sections underwent deparaffinisation and rehydration before antigen retrieval in pH 6 citrate buffer for 1 h in a steamer. Following blocking for non-specific binding, the sections were then labelled with beta galactosidase (1:100 Molecular Probes Cat# A-11132, RRID:AB_221539), Avidin 488 for mast cells (1:100 Life Technologies, Catalog#: A213370), anti-Mac2 for macrophages (1:100, CEDARLANE Cat# CL8942AP, RRID:AB_10060357), anti-CD86 for M1 macrophages (1:100, Santa Cruz Biotechnology Cat# sc-28,347, RRID:AB_627200) or anti-CD206 for M2 macrophages (1:100, Abcam Cat# ab64693, RRID:AB_1523910), anti-NK-1R (1:100, Santa Cruz Biotechnology Cat# sc-365,091, RRID:AB_10847681), anti-troponin T for cardiomyocytes (1:100, Thermo Fisher Scientific Cat# MA5–12960, RRID:AB_11000742) and anti-PDGFRA for cardiac fibroblasts (1:100, Thermo Fisher Scientific Cat# PA5–16571, RRID:AB_10981626). Mac2, cardiac troponin T and PDGFRA labelling were visualised using Alexa Fluor 568-goat anti-rat secondary antibody (1:100, Thermo Fisher Scientific Cat# A-11077, RRID:AB_2534121), CD86 and NK-1R labelling were visualised using Alexa Fluor 488-goat anti-rabbit secondary antibody (1:100, Thermo Fisher Scientific Cat# A-11008, RRID:AB_143165) and CD206 was visualised using Alexa Fluor 647-donkey anti-rabbit secondary antibody (1:100, Thermo Fisher Scientific Cat# A32795TR, RRID:AB_2866496). All sections were labelled with DAPI (Sigma Aldrich) for nuclei identification, and cover-slipped using VectaShield (Vector). The sections were then visualised and imaged using an EVOS M5000 fluorescence microscope (Life Technologies). For mouse primary cardiac fibroblast immunolabelling, cells were cultured on sterile coverslips and fixed in 4% PFA for 15 min. The cells were blocked in blocking buffer before being labelled with anti-NK-1R (1:100, Santa Cruz Biotechnology Cat# sc-365091, RRID:AB_10847681). Alexa Fluor 488-goat anti-rabbit secondary antibody (1:100, Thermo Fisher Scientific Cat# A-11008, RRID:AB_143165) was used to visualise the NK-1R. Phalloidin 647-conjugated antibody (1:100, Life Technologies, Cat#: A32795) was used to visualise the F-actin cytoskeleton. The cells also were labelled with DAPI (Sigma Aldrich) for nuclei identification and mounted using VectaShield (Vector). The sections were visualised using an EVOS M5000 fluorescent microscope. Avidin^+^, Mac2^+^CD86^+^ and CD206^+^ quantification was presented as cells per tissue section unit area. The experimental details provided conforms with the *BJP* Guidelines (Alexander et al., 2018).

### 2.10 | Cellular NK-1R distribution

NK-1R labelled mouse fibroblasts treated with NG, HG and HG + GR73632 (300 nM) were analysed using ImageJ2 (Rueden et al., 2017). Briefly, for each image, the cellular NK-1R distribution was analysed by measuring the pixel intensity along the cell body at 10-μm intervals, starting from the edge of the nucleus. This measurement process was repeated three times by rotating the image at 90°, creating a robust four directional average pixel intensity at 10-μm intervals. Ten cells per group were analysed.

### 2.11 | Terminal deoxynucleotidyl transferase dUTP nick end labeling (TUNEL)

5-μm formalin fixed LV sections underwent deparaffinisation and rehydration before being processed for TUNEL according to the manufacturer protocol (Promega). The sections also were labelled with DAPI (Sigma Aldrich) for nuclei identification and mounted using VectaShield (Vector). LV sections were visualised using an EVOS M5000 (Life Technologies) fluorescent microscope. Cells with TUNEL positive labelling co-localised with DAPI were considered dying cells and counted across the entire LV section.

### 2.12 | 3-Dimensional light sheet microscopy

Whole mouse hearts from WT, Lepr^db/db^ + saline, Lepr^db/db^ + GR73632 mice were dissected and major blood vessels removed, whilst keeping the atria intact. Tissue processing and labelling were performed according to the Miltenyi Biotech lightsheet microscopy tissue staining protocol. Briefly, hearts were fixed in 4% paraformaldehyde overnight before undergoing permeabilisation, dehydration, depigmentation and rehydration. Next, the hearts were incubated in staining solution containing mouse AlexaFluor647-conjugated SP (1:100, Bio-Rad Cat# 8450–0505, RRID:AB_2200292) primary antibody or mouse AlexaFluor647-conjugated troponin T antibody (1:500, Miltenyi Biotec Cat# 130-133-436, RRID:AB_3664263) for 7 days in a MACSmix tube rotator at 37°C. Next, the hearts were washed and dehydrated before undergoing a tissue clearing process using the MACS Clearing Kit. The hearts were then kept in MACS Imaging solution before images were acquired using a MACS UltraMicroscope.

### 2.13 | Quantitative polymerase chain reaction

RNA from LV or cultured mouse cardiac fibroblasts was extracted using the Qiagen RNA Extraction kit (Qiagen) and converted into complementary DNA (cDNA) using SuperScript^™^ IV VILO^™^ Master Mix (Life Technologies) according to the manufacturers’ instructions. Genes of interest such as *Col1a1*, Col3a1, Tac1, Tacr1 and *Gapdh* were then probed using ready-made TaqMan-based assays (Applied Biosystem) and amplified using QuanStudio^™^ 3 Real Time PCR System (Applied Biosystem). The gene amplification was analysed and quantified using the Design&Analysis 2 (DA2) software. The data was normalised to *Gapdh* as a house-keeping gene and presented as 2^−ΔCt^ (relative expression).

### 2.14 | Proteomics

Proteomic profiling was conducted by the IDeA National Resource for Quantitative Proteomics at the University of Arkansas for Medical Sciences. Briefly, the LV from WT, Lepr^db/db^ + saline and Lepr^db/db^ + GR73632 mice were reduced, alkylated, and purified by chloroform/methanol extraction prior to digestion with sequencing grade modified porcine trypsin (Promega, Madison, WI, USA). Tryptic peptides were then separated by a reverse phase Ion-Opticks-TS analytical column (25 cm × 75 μm with 1.7-μm C18 resin), supported by an EASY-Spray nano-source, and stabilised with a Heater THOR Controller (Ion-Opticks) at 60°C. Peptides were trapped and eluted from PepMap Neo, 300 μm × 5 mm Trap using a Vanquish Neo UHPLC nano system (Thermo Scientific), which kept the samples at 11°C before injection. Peptides were eluted at a flow rate of 0.350 μl min^−1^ using a 35-min gradient from 98% Buffer A (0.1% formic acid, 0.5% acetonitrile in water): 2% Buffer B (80% acetonitrile, 20% water, 0.1% formic acid) to 94.5:5.5 at 0.1 min to 56:44 at 27.1 min, followed by a column wash of 45:55 at 29.7 min to 1:99 at 35 min, followed by equilibration back to 98:2. Eluted peptides were ionised by electrospray (2.5 kV) followed by mass spectrometric analysis on an Orbitrap Astral mass spectrometer (Thermo Scientific). Precursor ion spectra were acquired from 380–980 mass per charge (*m*/*z*) at 240,000 resolutions.

### 2.15 | Bioinformatics

Following proteomic data acquisition, the raw data were processed and analysed using Express Analyst software. Proteins with low expression along with proteins with data completeness of 80% or more across all the samples were used for downstream analysis. This filtering threshold retained 6142 proteins from the initial of 6413 (95.8%) uniquely identified proteins. No technical or biological outliers were detected across the samples. Principal component analysis (PCA) was performed, and Differential Expression analysis was conducted using Limma design matrix. Protein intensity was transformed into Log2 measurement to generate a Gaussian-distributed dataset. False discovery rate (FDR)-adjusted *P* < 0.05, and fold change (FC) > ±1, was considered significant. Differential expression analysis was visualised as a heatmap. Unsupervised hierarchical clustering analysis was conducted with Pearson distance metric and average linkage. Volcano plots were then generated using Log (FDR) or Log (raw *P*-value) against Log2FC between groups. Kyoto Encyclopaedia of Genes and Genome (KEGG) pathway analysis, and Gene Ontology (GO) for biological processes, molecular mechanism and cellular component terms analysis were conducted between groups.

### 2.16 | Data and analysis

All groups generated in this study were of equal size, and analysis was performed in a randomised and blinded manner. Statistical analysis was undertaken for studies with group size of at least n = 5. The one case where we performed statistical analysis with less than n = 5, was for proteomics where n = 4. These results can be considered as preliminary. All grouped data were expressed as mean ± SD or SEM as appropriate. Group data comparisons were made by Student’s *t*-test, or one-way ANOVA for comparison of three or more groups. Tukey post-hoc test was used to analyse intergroup comparisons when ANOVA analysis identified an F-statistic of *P* < 0.05. Statistical significance was taken at *P* < 0.05. All group sizes used in the analysis were the number of independent values. The only instance where data were normalised was via log transformation for concentrations of SP and GR73632 treatments in Figure S2, which is standard practice. All experimental design and analysis complied with *British Journal of Pharmacology* recommendations and requirements on experimental design and analysis (Curtis et al., 2018).

### 2.17 | Materials

Aprepitant (Cat# 6486), GR73632 (Cat#1669) and L732138 (Cat#0868) were purchased from Tocris Biosciences. Details of other materials and suppliers are provided in specific subsections in Methods.

### 2.18 | Nomenclature of targets and ligands

Key protein targets and ligands in this article are hyperlinked to corresponding entries in https://www.guidetopharmacology.org, and are permanently archived in the Concise Guide to PHARMACOLOGY 2023/24 (Alexander et al., 2023).

## 3 | RESULTS

### 3.1 | Cardiac SP did not change in Lepr^db/db^ mice

**FIGURE 1:**
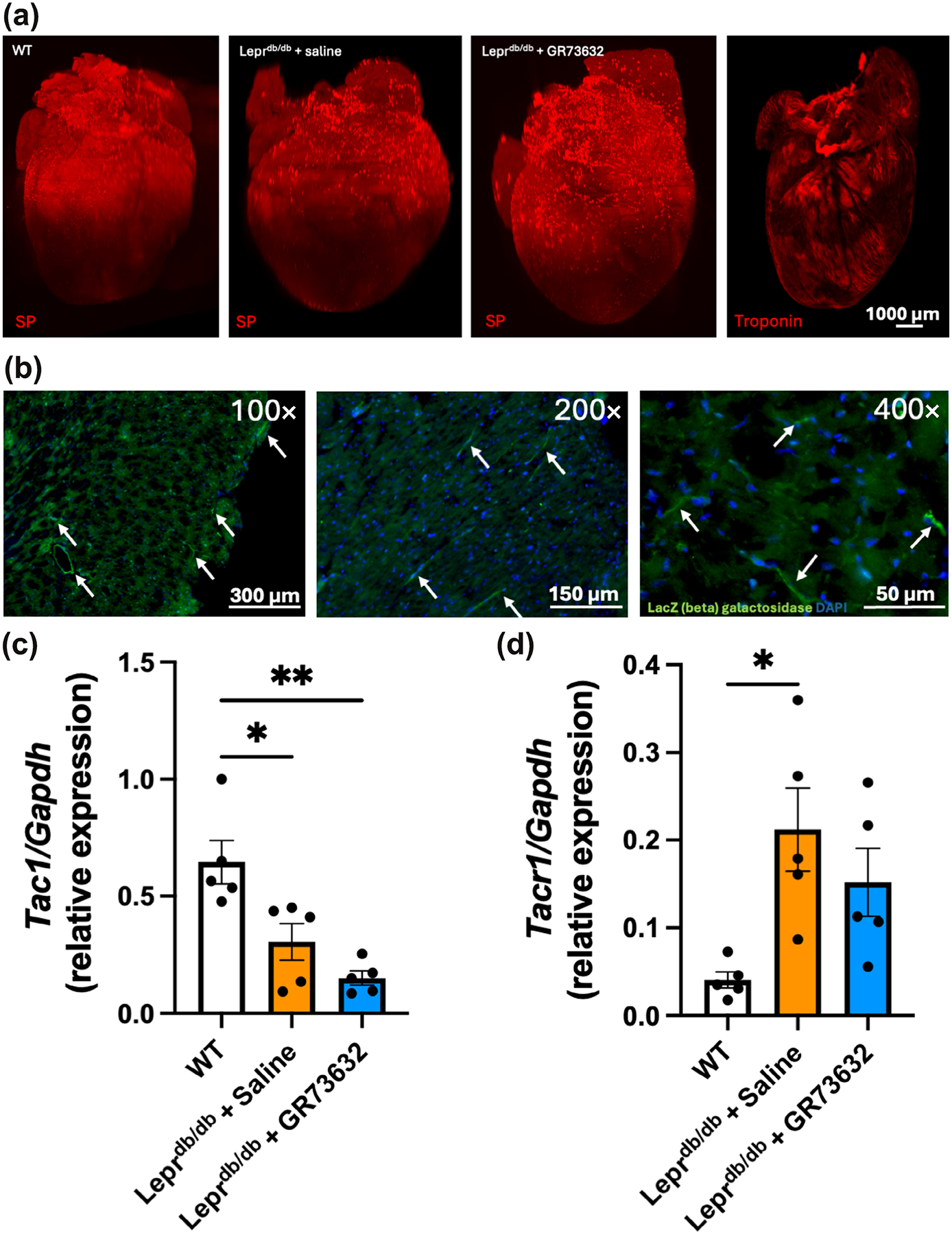
Substance P (SP) does not change in Lepr^db/db^ mouse hearts. (a) SP distribution in the heart of wild type (WT) (n = 3), Lepr^db/db^ + saline (n = 3) and Lepr^db/db^ + GR73632 (n = 3) mice; (b) LacZ (beta galactosidase) labelling for *Tacr1* in the mouse left ventricle (arrows) (n = 5); (c) *Tac1*; and (d) *Tacr1* mRNA levels in the left ventricular (LV) of WT (n = 5), Lepr^db/db^ + saline (n = 5) and Lepr^db/db^ + GR73632 (n = 5) mice. All values are mean ± SEM, *P < 0.05, **P < 0.01.

To gain a better understanding of SP distribution, diabetic hearts were imaged using 3-dimensional light sheet microscopy. Visually, SP appeared to be slightly more abundant in the Lepr^db/db^ mouse heart compared with WT, and more abundant again in Lepr^db/db^ with GR73632 (Figure 1a). This labelling was performed on three separate hearts, with the results being consistent each time. Next, *Tacr1* beta galactosidase (LacZ) reporter mice were used to identify expression of the NK-1R in the LV and demonstrated the expression of *Tacr1* on multiple cell types (Figure 1b). The presence of the NK-1R was further confirmed by the co-localisation of NK-1R antibody labelling with cardiomyocytes (Troponin T^+^), fibroblasts (PDGFRA^+^) and macrophages (Mac2^+^) (Figure S1). In contrast to SP protein, SP mRNA (*Tac1*), was significantly decreased in both Lepr^db/db^ + saline and Lepr^db/db^ + GR73632 compared with WT (Figure 1c), with no difference between the Lepr^db/db^ groups. *Tacr1* mRNA was increased significantly in the LV from Lepr^db/db^ + saline, but not in Lepr^db/db^ + GR73632, compared with WT (Figure 1d).

### 3.2 | GR73632 activate the NK-1R in cardiac fibroblasts

As a G-protein-coupled receptor, NK-1R activation can be measured through intracellular calcium as a second messenger. SP and the NK-1R agonist GR73632 induced a concentration-dependent increase in intracellular calcium in human cardiac fibroblasts. Pretreatment of the cells with the NK-1R antagonist, aprepitant, prevented a response to both SP and GR73632 (Figure S2).

### 3.3 | NK-1R activation improved cardiac function in Lepr^db/db^ mice

To determine whether NK-1R activation prevented or reversed specific cardiac remodelling parameters, 12 weeks (age at onset of GR73632 treatment) and 16 weeks of age (experimental end-point) Lepr^db/db^ mice were compared. Biometric data revealed that both 12-and 16-week-old, Lepr^db/db^ mice had significantly higher body weight in comparison to the age-matched WT mice (Table S1). At 16 weeks of age, Lepr^db/db^ mice had significantly lower LV, RV and lung index compared with the age-matched WT mice. Only right kidney index was increased in 12-week-old Lepr^db/db^ mice compared with the age-matched WT mice. No significant differences were observed in the biometric data between 12- and 16-week-old Lepr^db/db^ mice. There were no significant differences in LV end diastolic pressure between 12-week-old Lepr^db/db^ and WT mice, or between 16-week-old Lepr^db/db^ and WT mice (Figure S3a). However, there was a significant increase in LV end diastolic pressure in 16-week-old Lepr^db/db^ mice when compared with the 12-week-old Lepr^db/db^ mice. Rate of relaxation (minimum dP/d*t*) was significantly decreased in 12-week-old Lepr^db/db^ mice when compared with the age-matched WT mice, whereas no significant difference was observed between 16-week-old Lepr^db/db^ and WT mice (Figure S3B). Similarly, no significant difference was observed between 16-week-old Lepr^db/db^ compared with 12-week-old Lepr^db/db^ mice. The time constant, *Tau*, revealed that Lepr^db/db^ mice had increased cardiac stiffness compared with WT mice in both age groups (Figure S3c). However, no significant difference was observed between 16-week-old Lepr^db/db^ compared with 12-week-old Lepr^db/db^ mice. No significant differences were observed in LV end diastolic volume and ejection fraction between Lepr^db/db^ and WT mice across the age groups (Figure S3D&E). Although there was no significant difference in developed pressure between the 12-week-old Lepr^db/db^ and age-matched WT mice, there was a significant increase in 16-week-old Lepr^db/db^ mice compared with the age-matched WT mice (Figure S3f). No significant difference observed between 16-week-old Lepr^db/db^ compared with 12-week-old Lepr^db/db^ mice. No significant differences were observed for rate of contraction (maximum dP/d*t*) between Lepr^db/db^ and WT mice across the age groups (Figure S3g). There was a decrease in stroke volume and cardiac output in the 12-week-old Lepr^db/db^ mice when compared with the age-matched WT mice (Figure S3h,i). No significant difference was observed between the 16-week-old Lepr^db/db^ and WT mice. Interestingly, cardiac output was significantly decreased in 16-week-old WT mice when compared with 12-week-old WT mice. Although not significant, stroke work tended to decrease in 12-week-old Lepr^db/db^ mice and increase in 16-week-old Lepr^db/db^ mice when compared with their respective age-matched WT mice (Figure S3j). Stroke work was significantly decreased in the 16-week-old WT mice when compared with the 12-week-old WT mice. No significant difference was observed in heart rate for 12- and 16-week-old Lepr^db/db^ mice when compared with age-matched WT mice (Figure S3k). Whilst not statistically significant, collagen volume fraction tended to increase in 12-week-old Lepr^db/db^ mice compared with the age-matched WT mice, and there was significant increase in collagen volume fraction in 16-week-old Lepr^db/db^ mice compared with the age-matched WT mice. However no significant difference was observed between 16-week-old Lepr^db/db^ compared with 12-week-old Lepr^db/db^ mice (Figure S3l).

**FIGURE 2:**
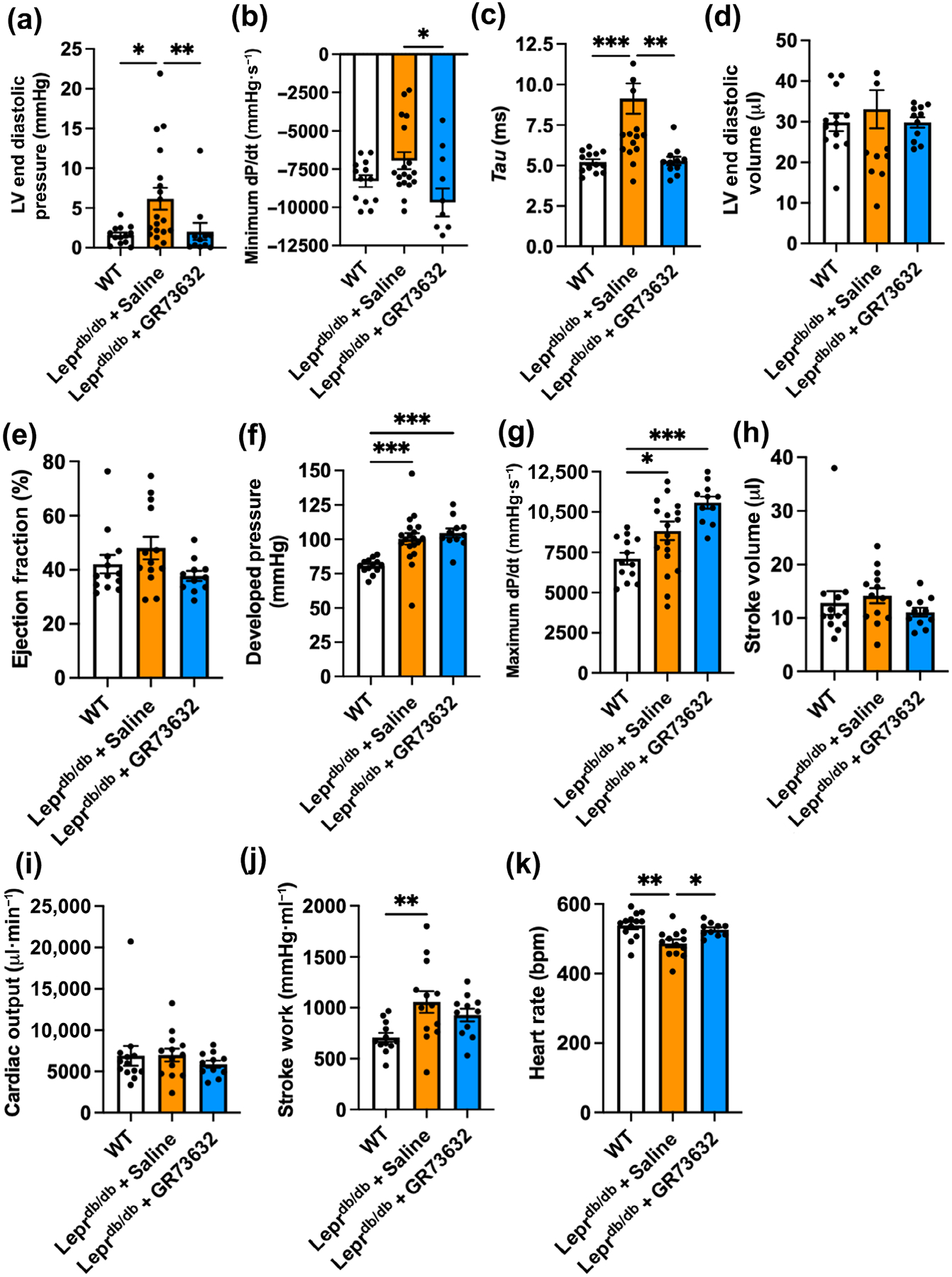
GR73632 treatment improves cardiac function in Lepr^db/db^ mice. (a) Left ventricle (LV) end diastolic pressure; (b) minimum dP/dt; (c) *Tau*; (d) LV end diastolic volume; (e) ejection fraction; (f) developed pressure; (g) maximum d*P*/d*t*; (h) stroke volume; (i) cardiac output; (j) stroke work; and (k) heart rate for wild type (WT) (n = 13), Lepr^db/db^ + saline (n = 19) and Lepr^db/db^ + GR73632 (n = 11) mice. All values are mean ± SD, *P < 0.05, **P < 0.01, ***P < 0.001.

Having established similarities and differences in disease progression in Lepr^db/db^ mice, we then evaluated the effect of NK-1R activation with GR73632. Biometric data revealed that at the 16 weeks of age timepoint, there was a significant increase in body mass in the Lepr^db/db^ + saline and Lepr^db/db^ + GR73632 groups compared with WT (Table 1). There was a significant decrease in the LV, RV and lung index between the Lepr^db/db^ groups and WT mice. However, there were no significant differences in these parameters between Lepr^db/db^ + saline and Lepr^db/db^ + GR73632 groups. No significant differences were observed in left and right kidney index across the groups. Pressure–volume catheterisation was performed to determine the effects of NK-1R activation on cardiac function in 16-week-old Lepr^db/db^ mice. Lepr^db/db^ + saline mice showed a significant increase in LV end diastolic pressure compared with WT, and this was prevented by GR73632 (Figure 2a). Although not statistically significant, rate of relaxation (minimum d*P*/d*t*) tended to be decreased in the Lepr^db/db^ + saline group, compared to WT, and GR73632 significantly increased the rate of relaxation compared with Lepr^db/db^ + saline mice (Figure 2b). The time constant, *Tau*, revealed that Lepr^db/db^ + saline mice had increased cardiac stiffness compared with WT mice (Figure 2c). GR73632 treatment reversed this increase in *Tau*. No significant differences in LV end diastolic volume or ejection fraction were observed across the groups (Figure 2d,e). Developed pressure and maximum rate of contraction (maximum d*P*/d*t*) were significantly increased in both Lepr^db/db^ + saline and Lepr^db/db^ + GR73632 groups compared with WT (Figure 2f,g) but were not different from each other. No differences were observed for stroke volume and cardiac output across the groups (Figure 2h,i). However, we did observe a significant increase in stroke work in the Lepr^db/db^ + saline group, with GR73632 tending to decrease this parameter, albeit not significantly (Figure 2j). We observed a significant decrease in heart rate for the Lepr^db/db^ + saline group, whereas GR73632 maintained normal rate (Figure 2k). We also measured wall-to-lumen ratio for intracoronary vessels ranging in size from <2000 to >10000 μm^2^ and found no statistically significant differences across the groups (Figure S4).

### 3.4 | Activation of the NK-1R reduced cardiac fibrosis and apoptosis in Lepr^db/db^ mice

**FIGURE 3:**
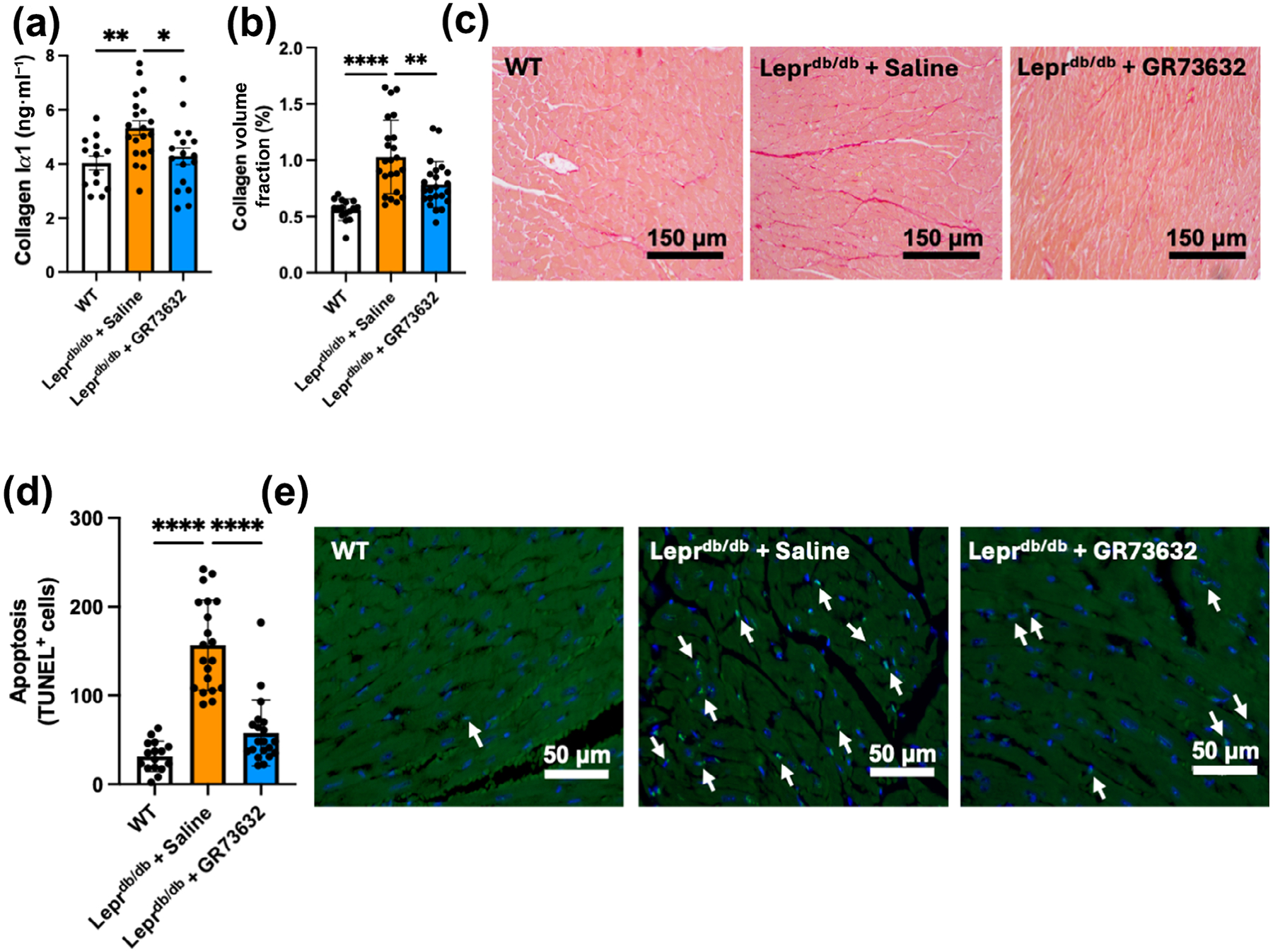
GR73632 treatment ameliorates cardiac fibrosis and apoptosis in Lepr^db/db^ mice. (a) Collagen I*α*1; (b) collagen volume fraction with; (c) representative images of picrosirius red staining for wild type (WT) (n = 13), Lepr^db/db^ + saline (n = 20) and Lepr^db/db^ + GR73632 (n = 17); (d) apoptosis with; (e) Representative images of transferase dUTP nick end labelling (TUNEL) positive staining for WT (n = 16), Lepr^db/db^ + saline (n = 20) and Lepr^db/db^ + GR73632 (n = 20). All values are mean ± SEM (a) and mean ± SD (b, d), *P < 0.05, **P < 0.01, ****P < 0.0001.

Next, the effects of NK-1R activation on cardiac fibrosis and cell death were studied. The Lepr^db/db^ + saline group showed a significant increase in collagen Iα1 content and collagen volume fraction compared with WT, with activation of the NK-1R with GR73632 significantly preventing the increase in both parameters (Figure 3a–c). The reverse approach of NK-1R antagonism with L732138 had no effect on collagen volume fraction for Lepr^db/db^ + saline mice (Figure S5). Lepr^db/db^ + saline mice also showed significant TUNEL positive labelling, an indication of significant cardiac apoptosis (Figure 3d,e). GR73632 significantly reduced the number of TUNEL positive cells.

### 3.5 | NK-1R activation reduced collagen, chemokine and cytokine release by cardiac fibroblasts under high glucose conditions

**FIGURE 4:**
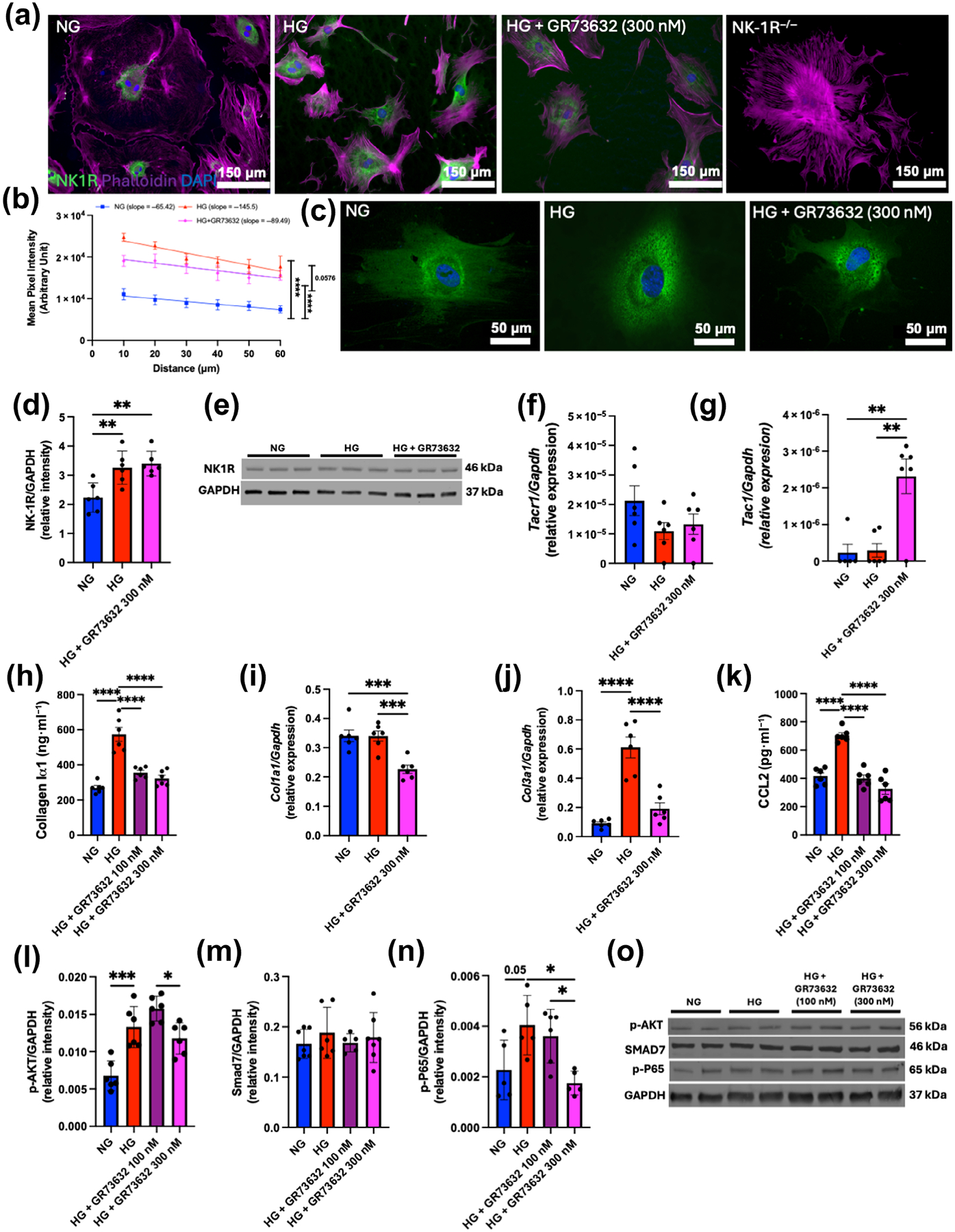
GR73632 reduces collagen release by cardiac fibroblasts exposed to high glucose (HG). (a) Immunofluorescence labelling of the NK-1R; (b) NK-1R cellular distribution with; (c) Representative NK-1R distribution images in mouse cardiac fibroblasts treated with normal glucose (NG), HG and HG + GR73632 (n = 10 per group); (d,e) NK-1R levels and the representative blot; (f) *Tacr1*; (g) *Tac1* mRNA levels; (h) collagen I*α*1 release into the media; (i) *Col1a1*; (j) *Col3a1* mRNA levels; (k) CCL2 release; (l–o) p-AKT, Smad7, p-P65 protein levels and the representative blots in NG, HG and HG + GR73632 treated mouse cardiac fibroblasts (n = 5–6 per group). All values are mean ± SEM, *P < 0.05, **P < 0.01, ***P < 0.001, ****P < 0.0001.

The direct effects of NK-1R activation were studied in vitro using primary cardiac fibroblasts cultured in HG media. The presence of the NK-1R on cardiac fibroblasts across the treatment groups was confirmed by immunofluorescence (Figure 4a). This labelling was performed on three separate fibroblast cultures, with consistent results each time. Negative NK-1R labelling in NK-1R^−/−^ fibroblasts confirmed antibody specificity. In WT mouse cardiac fibroblasts, NK-1R labelling showed a strong peri-nuclear localisation regardless of treatment condition (Figure 4b,c). However, peri-nuclear NK-1R levels were greater with HG than NG (Figure 4b). NK-1R labelling was higher overall, and significantly greater across the entirety of the cell under HG conditions (Figure 4b–e). Whereas the immunofluorescent (Figure 4b) and western blot (Figure 4d) data were generally in agreement with both showing higher NK-1R levels with HG, there was a small discrepancy with immunofluorescence indicating a small, but significant, decrease in NK-1R levels with GR73632, whereas western blotting did not detect a difference. Full NK-1R western blots are shown in Figure S6a. Of note, the slope for the localisation data in Figure 4b was greater in the HG group compared to NG and HG + GR73632, indicating that less receptor was translocated to the distal portions of the cell relative to the peri-nuclear region. Regardless, there was more NK-1R throughout the cell under HG conditions. Interestingly, there were no differences in *Tacr1* mRNA levels across groups (Figure 4f). Whilst we did not observe a change in *Tac1* gene transcription for HG compared with NG, GR73632 significantly increased *Tac1* under HG conditions (Figure 4g). Collagen Iα1 release into the media by cardiac fibroblasts was significantly increased by 24-h exposure to HG, whereas GR73632 at both 100 and 300 nM, prevented collagen Iα1 release (Figure 4h). At this chronic timepoint, although *Col1a1* mRNA did not change with HG (Figure 4i), *Col3a1* was significantly increased (Figure 4j). GR73632 significantly decreased both *Col1a1* and *Col3a1* relative to HG. Release of the pro-inflammatory chemokine CCL2 was significantly increased by HG, and GR73632 prevented this increase both at 100 and 300 nM (Figure 4k). We then probed for potential activated signalling pathways regulated by the NK-1R. We observed that HG significantly increased p-AKT in cardiac fibroblasts (Figure 4l,o). There was no significant effect of GR73632 on p-AKT relative to HG, although the 300-nM concentration of GR73632 did decrease p-AKT relative to the 100 nM concentration. Smad7 levels were unchanged across groups (Figure 4m,o). HG significantly increased p-P65 (NF-*κ*B), with GR73632 preventing this increase at the 300-nM concentration, but not the 100 nM (Figure 4n,o). Full western blots images are shown in Figure S6b.

### 3.6 | NK-1R activation opposed an inflammatory state

**FIGURE 5:**
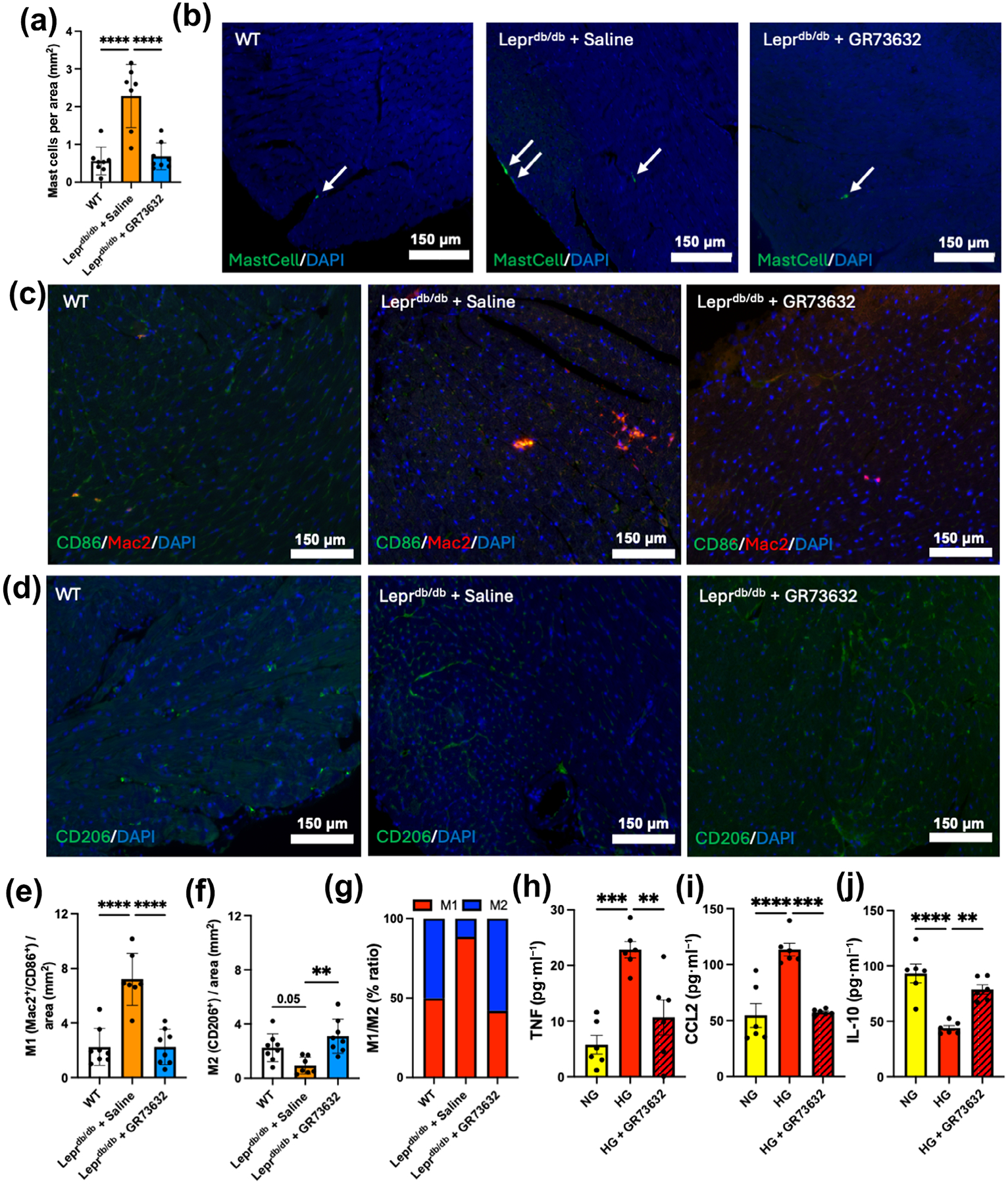
GR73632 treatment decreases mast cells and promotes anti-inflammatory macrophage phenotype in Lepr^db/db^ mouse left ventricle. (a) Mast cell numbers; (b) Avidin^+^ cardiac mast cells (arrows); (c) Mac2^+^/CD86^+^ macrophages and (d) CD206^+^ macrophages in wild type (WT) (n = 8), Lepr^db/db^ + saline (n = 8) and Lepr^db/db^ + GR73632 (n = 8) mice; (e) Mac2^+^/CD86^+^ macrophage numbers; (f) CD206^+^ macrophage numbers; (g) M1/M2 ratio; (h–j) TNF, CCL2 and IL-10 cytokine release by mouse bone marrow derived macrophages treated with normal glucose (NG), high glucose (HG) and HG + GR73632 (n = 6 per group). All values are mean ± SD (a, e and f). All values are mean ± SEM (h–j), **P < 0.01, ***P < 0.001, ****P < 0.0001.

We were interested in examining the effects of NK-1R activation on inflammation in the diabetic heart. The number of mast cells was significantly increased in LV from Lepr^db/db^ + saline mice, and normalised by GR73632 (Figure 5a,b). Similarly, inflammatory (M1) Mac2^+^CD86^+^ cardiac macrophages were significantly increased in the Lepr^db/db^ + saline group (Figure 5c,e), whereas anti-inflammatory CD206^+^ macrophages (M2) were significantly decreased (Figure 5d,f). This resulted in a higher M1/M2 ratio in Lepr^db/db^ + saline mice, indicative of an inflammatory state (Figure 5g). GR73632 reversed these cardiac macrophage phenotypes, resulting in restoration of a normal M1/M2 ratio. This phenotype reversal also was observed in BMM*θ*. The release of pro-inflammatory mediators TNF and CCL2 by BMM*θ* was significantly increased by HG and significantly down-regulated by GR73632 (Figure 5h,i). Moreover, the anti-inflammatory cytokine IL-10 was significantly down-regulated by HG, with this decrease prevented GR73632 treatment (Figure 5j).

### 3.7 | Proteomic assessment in the diabetic mouse heart

**FIGURE 6:**
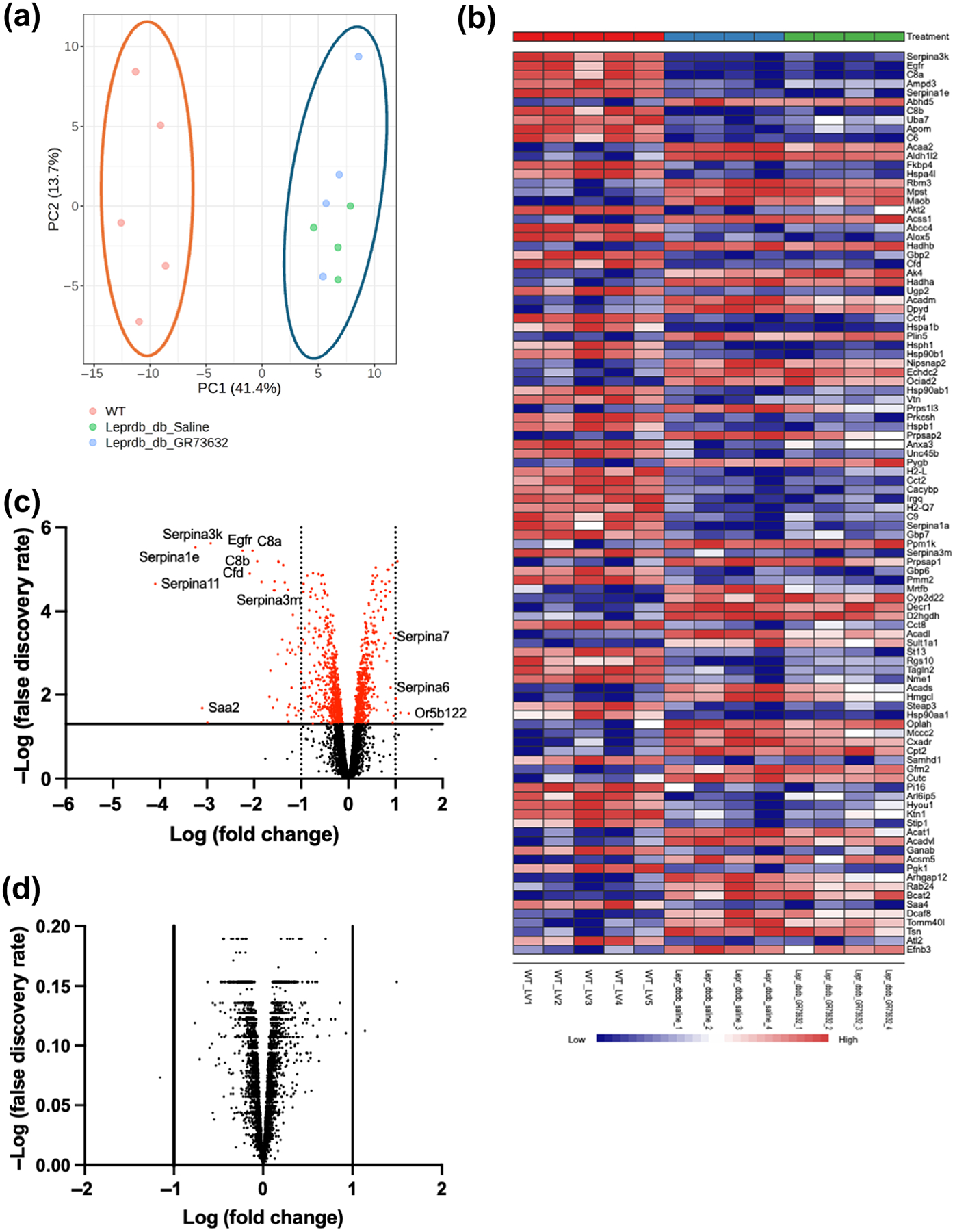
Left ventricle proteome signatures for Lepr^db/db^ and wild type (WT) mice. (a) Separation of groups by unsupervised principal component analysis (PCA); (b) heatmap of unsupervised hierarchical clustering of the 100 most differentially expressed proteins (false discovery rate adjusted *P* < 0.05 and fold change [FC] > ±1); volcano plots of false discovery rate (adjusted) cardiac proteomics analysis of (c) Lepr^db/db^ + saline (n = 4) versus WT (n = 5) mice and (d) Lepr^db/db^ + GR73632 (n = 4) versus Lepr^db/db^ + saline mice (n = 4); fold change (FC) > ±1.

Mass spectrometry-based proteomic profiling was performed on LV from WT, Lepr^db/db^ + saline and Lepr^db/db^ + GR73632 mice at 16 weeks of age. These results are considered preliminary as they are based on n = 4. PCA revealed that the LV proteomes from Lepr^db/db^ mice were distinct from the WT (Figure 6a, PC1 and PC2 with each explaining 41.4% and 13.7% of the variance, respectively). However, we did not observe a clear distinction between the proteomes of Lepr^db/db^ + saline and Lepr^db/db^ + GR73632 groups. Heat map representation of unsupervised hierarchical clustering of the top 100 differentially expressed proteins distinctly delineated the Lepr^db/db^ and WT proteomic profiles (Figure 6b). Using the adjusted *P*-value, we identified a total of 46 unique differentially expressed proteins between Lepr^db/db^ + Saline and WT (4 up-regulated and 42 down-regulated, Figure 6c). When comparing Lepr^db/db^ + GR73632 against Lepr^db/db^ + saline, no unique differentially expressed proteins were identified (Figure 6d).

**FIGURE 7:**
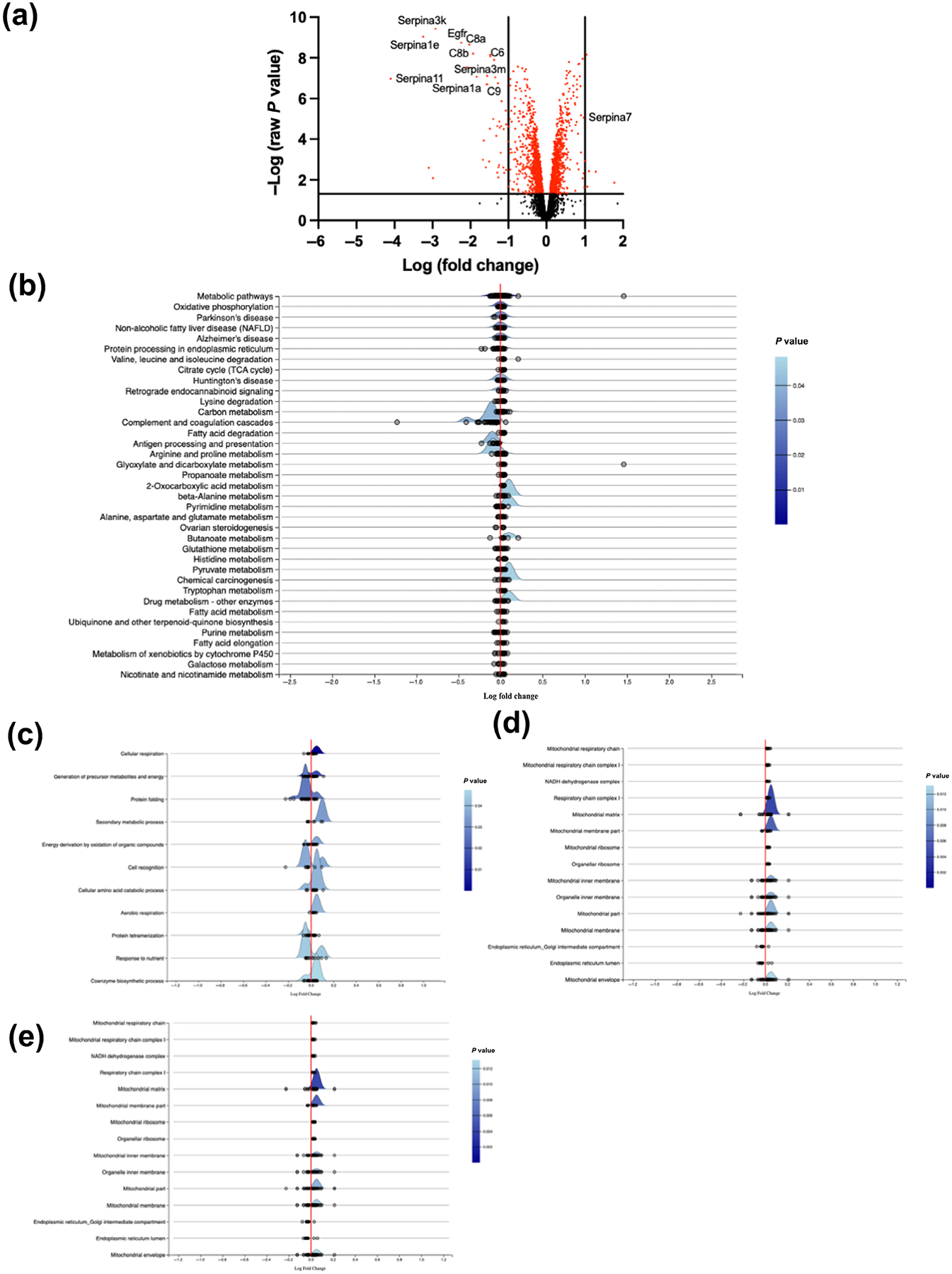
Proteomic profile for the Lepr^db/db^ mouse left ventricles. (a) Volcano plots of raw cardiac proteomics analysis of Lepr^db/db^ + saline versus wild type (WT), with fold change (FC) > 1; (b) Kyoto Encyclopaedia of Genes and Genome (KEGG) analysis pathway; (c) Gene Ontology (GO)-Biological Processes; (d) GO-Molecular Functions; and (e) GO-Cellular Compartment term analysis of Lepr^db/db^ + saline (n = 4) versus WT (n = 5).

Using the raw *P*-value, we identified a total of 49 unique differentially expressed proteins between Lepr^db/db^ + saline and WT (6 up-regulated and 43 down-regulated, Figure 7a). KEGG pathway analysis revealed complement and coagulation cascade proteins to be the most down-regulated compared to WT, in particular C8a and C9 with Log2FC values of −2.03 and −1.58, respectively (Figure 7b and Table S2). Serpina1a, Serpina1d and Serpina1e also were down-regulated with Log2FC values of −1.56, −0.96 and −3.25, respectively (Figure 7b and Table S2). Gene Ontology for Biological Processes identified Protein Folding as the most down-regulated cluster, whilst up-regulated proteins were more variable across clusters (Figure 7c). Molecular Mechanism and Cellular Component analysis revealed that Mitochondrial Matrix proteins were the most up-regulated proteins in Lepr^db/db^ + saline mice when compared with WT mice (Figure 7d,e).

**FIGURE 8:**
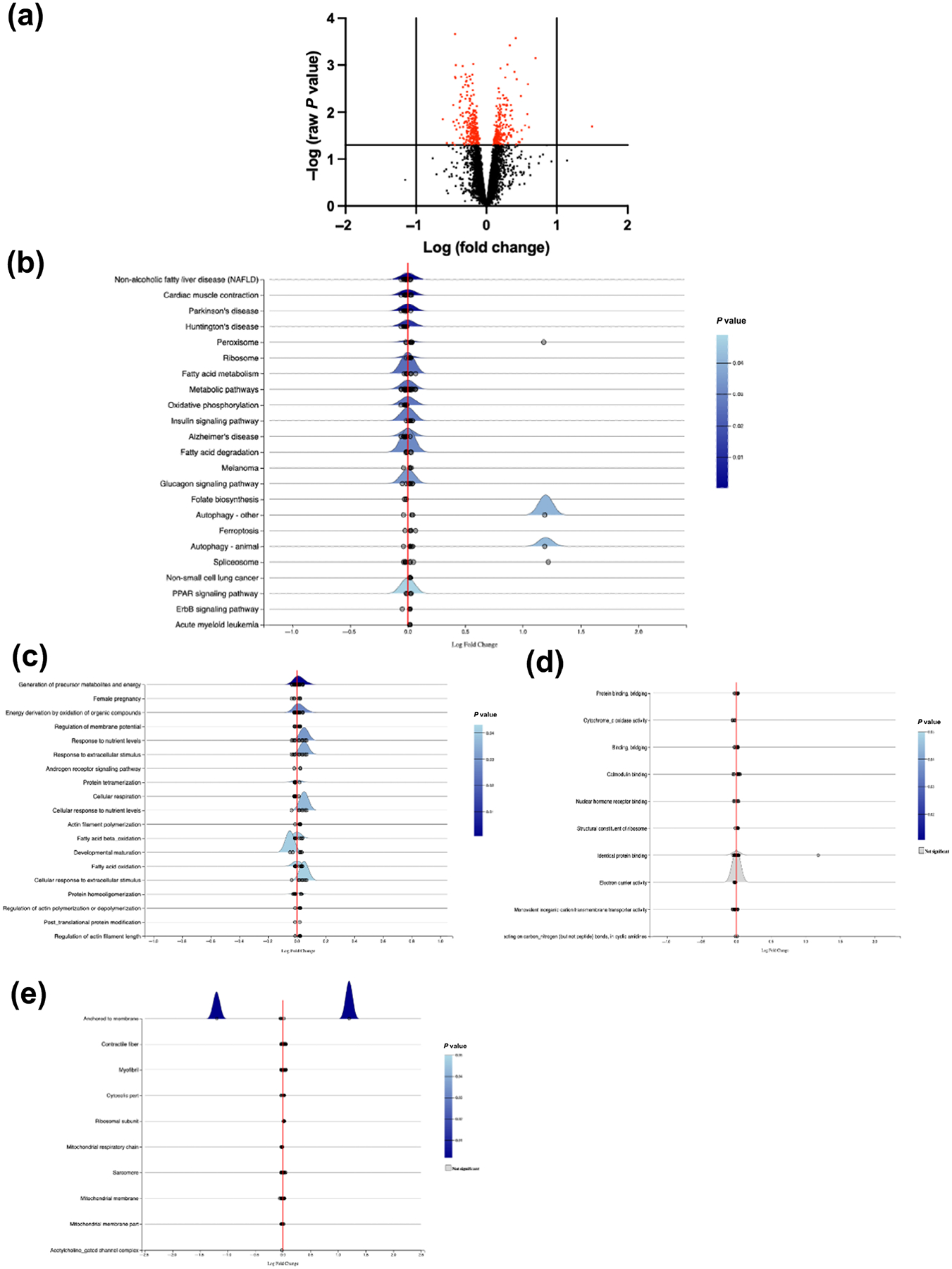
Proteomic profile for GR73632 treated Lepr^db/db^ mouse left ventricles. (a) Volcano plots of raw cardiac proteomics analysis of Lepr^db/db^ + GR73632 mice versus Lepr^db/db^, with fold change (FC) > 1; (b) Kyoto Encyclopaedia of Genes and Genome (KEGG) analysis pathway; (c) Gene Ontology (GO)-Biological Processes; (d) GO-Molecular Functions; and (e) GO-Cellular Compartment term analysis of Lepr^db/db^ + GR73632 (n = 4) mice versus Lepr^db/db^ + saline (n = 4).

Interestingly, we only observed one significantly up-regulated, but unidentified, protein between Lepr^db/db^ + GR73632 and Lepr^db/db^ + saline (Figure 8a). However, albeit not significant, KEGG analysis identified autophagy/cell death related proteins to be the most up-regulated in the LV of Lepr^db/db^ + GR73632 against Lepr^db/db^ + saline, including various pro-cell death proteins such as Atg9a and Bad with Log2FC values of 0.24 and 0.15 respectively, and anti-cell death proteins such as Akt2 with a Log2FC value of 0.13-fold (Figure 8b and Table S3). Gene Ontology for Biological Processes analysis showed Response to External Stimulus proteomes to be the most up-regulated across the identified clusters (Figure 8c and Table S4). Molecular Mechanism analysis showed no significant change across the clusters (Figure 8d), and Cellular Component analysis revealed only proteomes related to Membrane Anchors were down-regulated and up-regulated in Lepr^db/db^ + GR73632 when compared with Lepr^db/db^ + saline (Figure 8e and Table S5).

## 4 | DISCUSSION

To date, there are no effective treatment strategies to prevent or reverse T2DM-induced cardiac fibrosis and diastolic dysfunction, despite the increased mortality associated with the disease (Wong et al., 2014). Our previous studies established that replacing lost SP both in mice and non-human primates is an effective therapeutic strategy to target cardiac fibrosis and cardiac function in T2DM (Meléndez et al., 2023; Widiapradja et al., 2021). However, we had not identified the specific anti-fibrotic SP receptor. In this study, we demonstrate that NK-1R activation: (1) reduced cardiac fibrosis and improved cardiac function in the T2DM mouse; (2) reduced inflammation by decreasing mast cell numbers and promoting an anti-inflammatory macrophage phenotype in the T2DM mouse heart; and (3) prevented a HG-induced pro-fibrotic fibroblast phenotype.

We have previously described that a reduction in circulating SP in Lepr^db/db^ mice and T2DM monkeys occurs concomitantly with cardiac fibrosis, and that replacement SP reduced this fibrosis (Meléndez et al., 2023; Widiapradja et al., 2021). This led us to investigate candidates for the anti-fibrotic SP receptor. The NK-1R is the cognate receptor for SP and is encoded by the *Tacr1* gene (Levick et al., 2010). Using an antibody approach, we have previously shown that the NK-1R is expressed in the heart (Dehlin et al., 2013). In the current study we used both an antibody approach and a *Tacr1* reporter mouse, which confirmed NK-1R expression in the heart. Hence, we then treated Lepr^db/db^ mice with the selective NK-1R agonist, GR73632, and assessed cardiac function and fibrosis. Herein, we identified the NK-1R as the anti-fibrotic SP receptor because NK-1R activation reversed cardiac fibrosis. The reverse strategy of NK-1R blockade had no effect on cardiac fibrosis. NK-1R activation also reversed diastolic dysfunction in Lepr^db/db^ mice, as determined by LV end diastolic pressure, minimum rate of relaxation, and the time constant, *Tau*. That NK-1R activation could reverse remodelling and dysfunction has significant potential clinical application. Interestingly, the anti-fibrotic effect of GR73632 was achieved at less than one third of the SP dose that was used in our previous study (300 μg·kg^−1^·day^−1^ vs. 1 mg·kg^−1^·day^−1^). Several possibilities may account for this enhanced potency: (1) GR73632 has greater selectivity for the NK-1R (Regoli et al., 1988). Whereas SP has several hundred-fold greater potency at the NK-1R, it can also activate the NK-2R and NK-3R (Steinhoff et al., 2014), potentially diluting SP actions at the NK-1R. GR73632 has negligible activity at the NK-2R (Regoli et al., 1988); and (2) structurally, GR73632 is a modified version of the SP metabolite SP7–11 (amino acids 7 to 11, Table S6), thus, GR73632 may inherently be less susceptible to further metabolism, and additionally, has structural modifications to reduce degradation.

It is well established that hyperglycaemic conditions stimulate proliferation and pro-fibrotic phenotype conversion by fibroblasts (Aguilar et al., 2014; Fiaschi et al., 2014; Levick & Widiapradja, 2020; Román-Pintos et al., 2016; Shamhart et al., 2014). In our previous study, SP treatment prevented myofibroblast conversion and extracellular matrix release by mouse cardiac fibroblasts under HG conditions (Widiapradja et al., 2021). This included collagen I and III, as well as fibronectin and laminin. Herein, we demonstrate that isolated cardiac fibroblasts express the NK-1R and that GR73632 prevented collagen I release. Thus, the NK-1R is the anti-fibrotic SP receptor at the level of the fibroblast. Interestingly, at the time-point used in this study (24 h), *Col1a1* was not up-regulated by HG. *Col1a1* was likely up-regulated earlier in the time course; however, in our hands only *Col3a1* up-regulation was sustained.

Several potential mechanisms for NK-1R activation to oppose a HG-induced pro-fibrotic fibroblast phenotype were assessed. We first investigated AKT due to the protective actions of the NK-1R on cardiomyocytes under hypoxic conditions via AKT (Chen et al., 2015; Jubair et al., 2015). HG is known to increase AKT phosphorylation in both isolated neonatal and adult mouse cardiac fibroblasts, and was associated with excess collagen production via induction of ERK (Chen et al., 2015; Venkatachalam et al., 2008). Our findings with HG are consistent with this AKT activation. GR73632 did not alter HG-induced p-AKT. AKT activation is complicated, and it is possible that NK-1R activation may shift AKT signalling away from ERK-related pathways in fibroblasts to provide protection against a pro-fibrotic fibroblast phenotype, however, we did not assess this possibility. Because HG can induce TGF-β1 (Levick & Widiapradja, 2020), we wondered if NK-1R activation might up-regulate Smad7 as a negative regulator of Smad2/3 (Deng et al., 2024; Hanyu et al., 2001). However, this was not the case. Conversely, the 300-nM concentration, but not the 100-nM concentration of GR73632, inhibited P65 phosphorylation. This indicates that NK-1R activation blunts pro-inflammatory NK-κB signalling induced by HG. This was confirmed by the observation that the NK-*κ*B-regulated chemokine CCL2, was down-regulated by GR73632. What is interesting is that whereas both the 100- and 300-nM concentrations of GR73632 oppose a pro-fibrotic fibroblast phenotype, only the 300-nM concentration down-regulated NK-*κ*B signalling. Thus, the mechanisms seem to differ depending on concentration.

Another interesting observation was that NK-1R activation induced the up-regulation of *Tac1* in fibroblasts, albeit *Tac1* was detected at very low levels. Thus, as well as directly inhibiting pro-fibrotic and pro-inflammatory pathways, NK-1R activation may also induce protective autocrine pathways (i.e., SP synthesis), given that we have previously shown that SP opposes a pro-fibrotic fibroblast phenotype (Widiapradja et al., 2021). Conversely, no change in *Tacr1* was observed with HG. However, NK-1R protein was clearly increased in cardiac fibroblasts in response to HG. Spatially, we observed prominent peri-nuclear localisation of the NK-1R in the fibroblasts, which increased under HG conditions. HG also caused increased NK-1R throughout the cell. The change gradient for distribution of the NK-1R across the cell was more negative for HG compared to NG conditions indicating less relative translocation of the NK-1R to the outer areas of the cell relative to the per-nuclear region. Regardless, there was more NK-1R throughout the cell under HG conditions. The HG-induced increase in NK-1R levels is likely a protective mechanism to increase availability of the NK-1R for activation. Although we identified some subtle differences in distribution of the NK-1R under HG conditions, we cannot say whether this involves translocation to the cell membrane. SP and GR73632 maintained intracellular NK-1R localisation in rat brain ventral striatum synaptosomes (Arapulisamy et al., 2013) and mouse kidney epithelial cells (Roosterman et al., 2004), but whether this is also true for cardiac fibroblasts in our study is unclear. Regardless, NK-1R activation could be achieved and opposed a pro-fibrotic cardiac fibroblast phenotype.

T2DM-induced inflammation is heavily involved in the pathogenesis of cardiac fibrosis, and it involves mast cells and macrophages among other immune cells (Russo & Frangogiannis, 2016). In diabetes, wound healing is impaired because inflammation is sustained by a persistent pro-inflammatory M1 macrophage phenotype. Exogenous SP promotes the transition from pro-inflammatory to anti-inflammatory state, resolving inflammation and promoting proper wound healing (Leal et al., 2015; Lim et al., 2017; Montana & Lampiasi, 2016). We established that exogenous SP opposed the pro-inflammatory M1 phenotype, favouring the anti-inflammatory M2 phenotype in BMM*θ* cultures under HG conditions, as well as in the LV of T2DM Lepr^db/db^ mice (Widiapradja et al., 2021). In our current study, GR73632 inhibited Mac2^+^CD86^+^ pro-inflammatory macrophages, restoring a normal M1/M2 ratio. The ability of GR73632 to directly shift macrophage phenotype from M1 to M2 phenotype was confirmed using BMM*θ* exposed to HG. Interestingly, mast cell numbers in the LV of Lepr^db/db^ mice also were decreased with NK-1R activation. In the diabetic heart, mast cells play a significant role in cardiac fibrosis through chymase-induced TGF-β1 activation (Liu et al., 2009). Thus, part of the anti-fibrotic effects of the NK-1R involve inhibiting inflammation by several mechanisms. Whereas we demonstrate that activation of the NK-1R has direct effects on fibroblast and macrophage phenotype, it is also likely that cell crosstalk is involved. For example, we show that NK-1R activation on cardiac fibroblasts prevents release of the chemokine CCL2, which would be expected to have actions on macrophage recruitment. The involvement of crosstalk from other cells such as mast cells, endothelial cells or cardiomyocytes also may be a part of the overall anti-inflammatory/anti-fibrotic effects of NK-1R activation.

We also examined the wider effects of diabetes and NK-1R activation on the heart. Proteomic studies revealed an overall decrease in several protein groupings in the T2DM heart, including complement-related proteins, coagulation cascades and Serpin family proteins, which are serine/cysteine peptidase inhibitors. Of note, decreased Serpina1a is correlated with increased fibrosis in the liver, as well as the transcription of inflammatory genes such as *Tnf, Ccl2*, Ccl3, Ccl4 and Ccl5 (Park et al., 2025). We observed a similar trend in this study, in that the decrease in Serpina1a was accompanied with increased fibrosis in the diabetic heart. Somewhat surprisingly, despite clear effects on fibrosis and diastolic function, we did not observe significant differences in the proteome profiles between GR73632 treatment and saline in Lepr^db/db^ hearts. Several reasons could contribute to this: (1) GR73632 may affect phosphorylation of proteins that appeared in our analysis, which we did not assess; and/or (2) GR73632 affects low abundance proteins that did not appear in our analysis. Nevertheless, we do observe a non-significant decrease in proteins such as Cd59a and Ly6c1, indicating downregulation of inflammation in GR73632 treated Lepr^db/db^ mice, which is consistent with our data showing fewer inflammatory macrophages.

Another interesting observation was that light sheet microscopy suggested that there was no decrease in cardiac SP in Lepr^db/db^ mice. This is in contrast to our previous report that SP was reduced by roughly 50% in serum from Lepr^db/db^ mice at the same age as in the current study (Widiapradja et al., 2021), and plasma from diabetic non-human primates (Meléndez et al., 2023). Moreover, our finding is in disagreement with Ren and colleagues, who reported a decrease in SP in the heart of the STZ model of diabetes (Ren et al., 2011). Interestingly, despite no loss of cardiac SP in Lepr^db/db^ hearts, the transcription level of *Tac1* decreased significantly. It could be that this set of mice were on a slightly slower timeline than in our previous study, with the reduced *Tac1* not yet translating to reduced SP protein. Alternatively, there may not be a reduction in SP in the heart, but rather that SP is metabolised before reaching the serum, resulting in reduced serum levels of SP. Regardless of SP levels, exogenous activation of the NK-1R is beneficial.

Something that we did not investigate in this study but should be mentioned is the potential effect that NK-1R activation may have on adipose tissue and, thereby, indirectly fibrosis. NK-1R activation did not affect obesity per se, since body weight was not altered by GR73632, however, it could have affected adipocyte phenotype. Of itself, this is interesting, because mice with NK-1R deletion gain less weight is response to a high fat diet (Karagiannides et al., 2011), although this was due to reduced food intake. NK-1R-deficient mice had lower leptin and insulin levels, as well as unchanged adipokine and blood glucose levels compared with wild type mice on a high fat diet. Since these changes were likely related to reduced food intake, those findings may not be directly relatable to the Lepr^db/db^ mouse used in our study, where a loss of function mutation in the leptin receptor prevents satiety, leading to diabetes and obesity. We have shown previously that SP opposes a pro-inflammatory adipose macrophage phenotype in T2DM monkeys (Meléndez et al., 2023), suggesting that NK-1R activation may dampen inflammation in adipose tissue in diabetes, despite not altering obesity or blood glucose.

In summary, we report that selective NK-1R activation is effective at reversing fibrosis and improving diastolic function in diabetic hearts. The anti-fibrotic actions of NK-1R activation involve direct effects on cardiac fibroblasts to oppose a pro-fibrotic fibroblast phenotype. This likely involves down-regulation of NF-*κ*B and collagen genes. Activation of the NK-1R also reduced inflammation. This study identifies the NK-1R as the anti-fibrotic SP receptor. This finding has potential clinical relevance based on our previous findings that SP reduced fibrosis and improved cardiac function in T2DM non-human primates.

## Supplementary Material

supplementary file

Additional supporting information can be found online in the Supporting Information section at the end of this article.

## Figures and Tables

**TABLE 1:** Biometrics for 16-weeks-old WT, Lepr^db/db^ + saline and Lepr^db/db^ + GR73632 mice.

16-weeks-old	WT (mean ± SD)	Lepr^db/db^ + saline (mean ± SD)	Lepr^db/db^ + GR73632 (mean ± SD)
Body weight (gram)	30.5 ± 1.92	44.8 ± 7.43***	47.6 ± 5.65***
Left ventricle index^a^	3.83 ± 0.47	3.36 ± 0.39**	3.33 ± 0.29**
Right ventricle index^a^	1.17 ± 0.19	0.96 ± 0.15***	0.99 ± 0.12**
Lung index^a^	5.73 ± 0.60	4.65 ± 0.57****	4.61 ± 0.51****
Left kidney index^a^	6.84 ± 1.02	6.71 ± 1.15	6.56 ± 0.70
Right kidney index^a^	7.31 ± 1.08	7.01 ± 1.10	7.32 ± 0.83

a Normalised to tibia length (mg·mm^−1^).

** *P* < 0.01.

*** *P* < 0.001.

**** *P* < 0.0001 versus WT. n = 16–23 per group.

## Data Availability

The data that support the findings of this study are available from the corresponding author upon reasonable request. Some data may not be made available because of privacy or ethical restrictions.

## What is already known?

- Substance P loss leads to cardiac fibrosis and diastolic dysfunction in type 2 diabetes.
- Replacement substance P decreases cardiac fibrosis and improves diastolic function in type 2 diabetes.

## What does this study add?

- This study identified the NK-1R as the anti-fibrotic substance P receptor.
- The anti-inflammatory/anti-fibrotic actions of the NK-1R involve downregulation of NF-*κ*B.

## What is the clinical significance?

- This study identified a novel anti-fibrotic target for the treatment of diabetic cardiomyopathy.
- The NK-1R is translationally relevant since replacement substance P improved diabetic cardiomyopathy in a pre-clinical monkey model.

## Abbreviations:

HFpEF: heart failure with preserved ejection fraction
HG: high glucose
LA: lumen area
LV: left ventricle
NG: normal glucose
NK-1R: neurokinin-1 receptor
RV: right ventricle
SP: substance P
T2DM: type 2 diabetes mellitus
VA: vessel area
WLR: wall-to-lumen ratio
