# Supplementary material for: Neurokinin-1 receptor activation protects against cardiac fibrosis, inflammation and diastolic dysfunction in type 2 diabetic mice
Source: Br J Pharmacol. Author manuscript; Available in PMC 2026 May 12. (PMC13159039; doi:10.1111/bph.70440)
Supplement: supplementary file [file NIHMS2172611-supplement-supplementary_file.pdf]

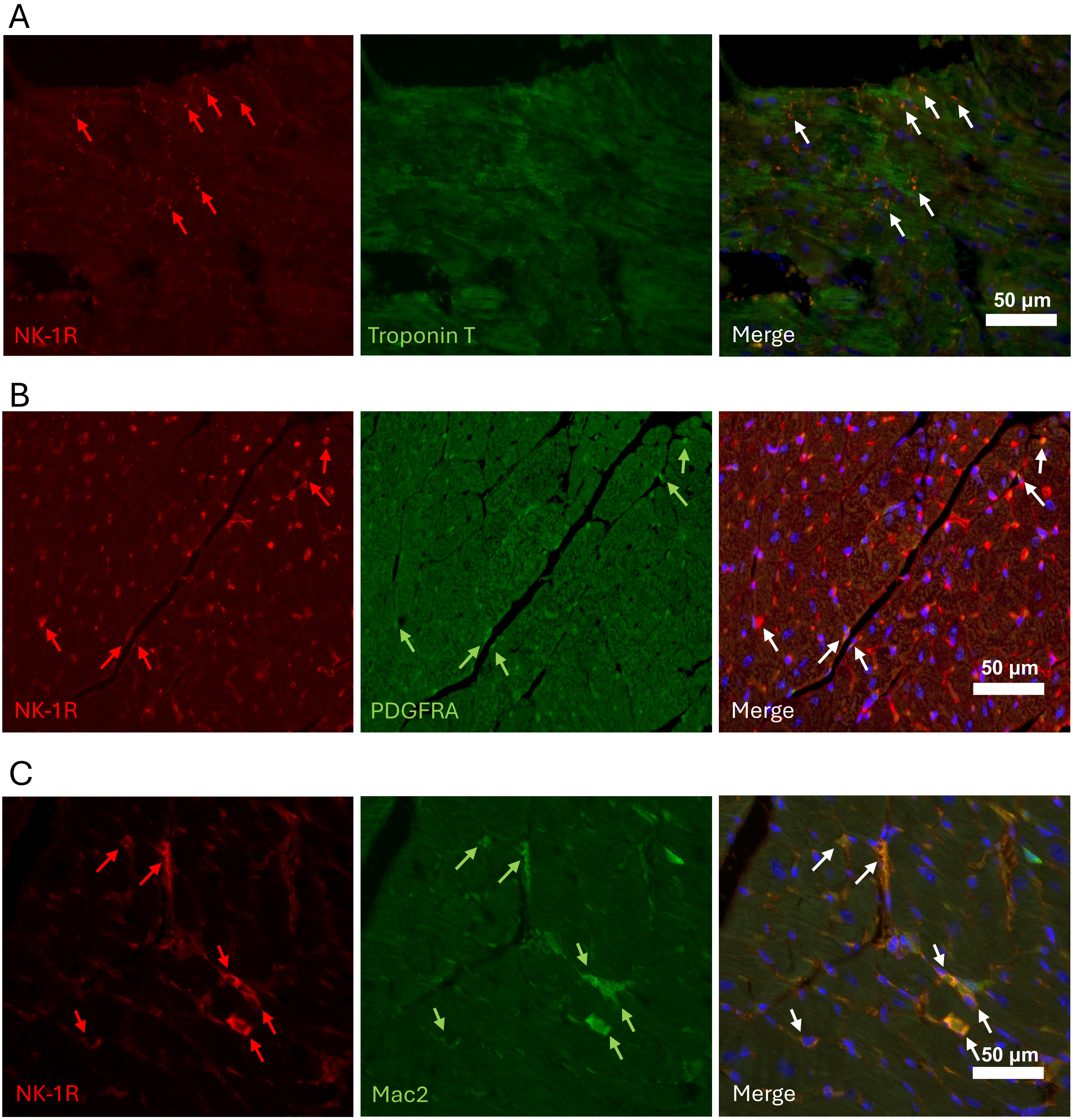

Supplemental Figure 1. Identification of the NK-1R presence in cardiac cells. NK-1R (red arrows) co-localization with (A) cardiomyocytes (Troponin T^{+}); (B) fibroblasts ($PDGFRA^{+}$, green arrows) and; (C) macrophages ($Mac2^{+}$, green arrows).

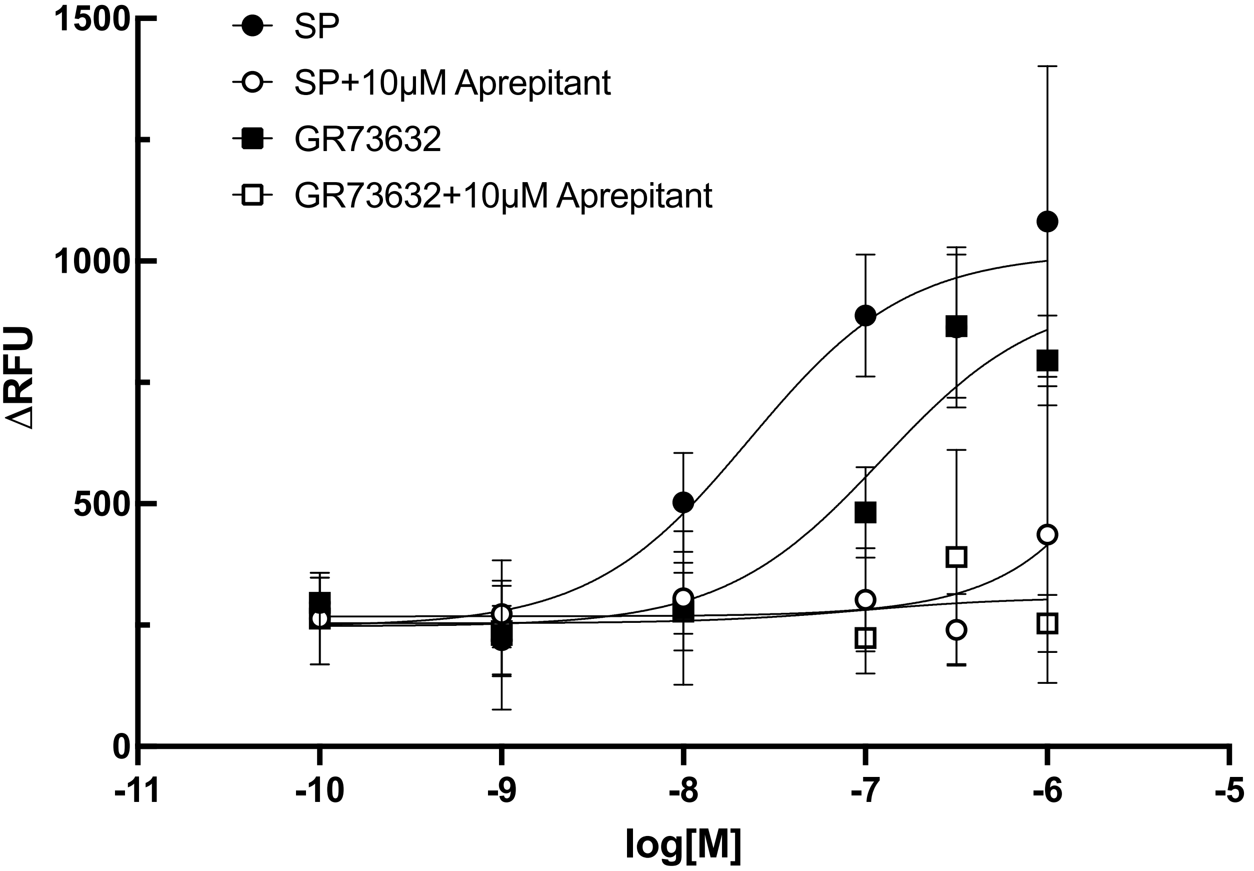

Supplemental Figure 2. NK-1R activation by SP and GR73632 in cardiac fibroblasts. NK-1R antagonist, Aprepitant prevents NK-1R activation by SP and GR73632. EC50 (SP): 2.308×10^{-8}M, EC50 (SP+10μM Aprepitant): 0.3M, EC50 (GR73632): 1.235×10^{-7}M, EC50 (GR73632+10μM Aprepitant): 9.488×10^{-8}M. n=12/group.

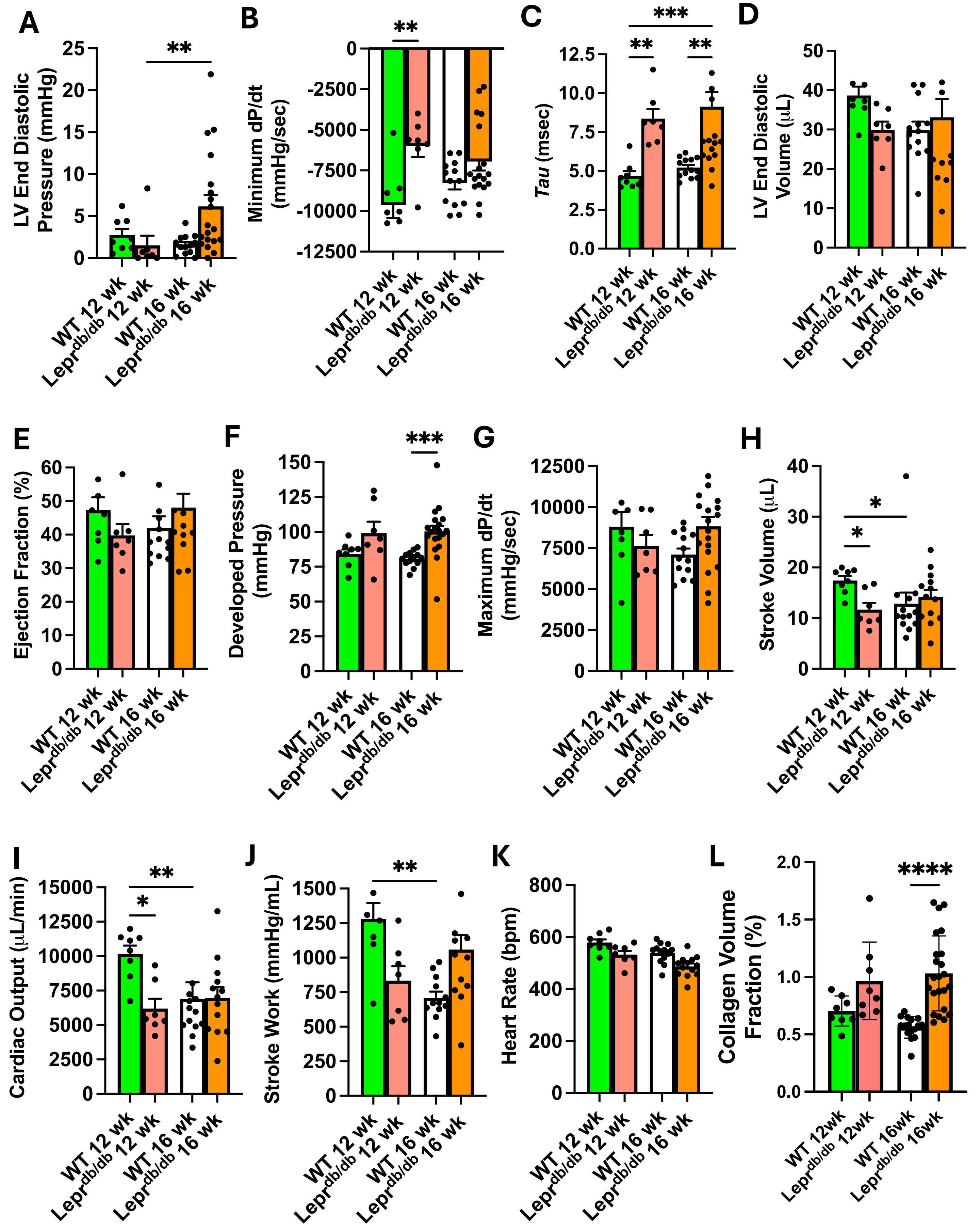

Supplemental Figure 3. Cardiac function for 12 week old and 16 week old WT and $Lepr^{db/db}$ mice. (A) Left ventricle (LV) end diastolic pressure; (B) Minimum dP/dt; (C) *Tau*; (D) LV end diastolic volume; (E) Ejection fraction; (F) Developed pressure; (G) Maximum dP/dt; (H) Stroke volume; (I) Cardiac output; (J) Stroke work; (K) Heart rate and; (L) Collagen volume fraction for 12 week old and 16 week old WT (n=8-13) and $Lepr^{db/db}$ (n=8-23) mice. All values are mean ± SD, *p<0.05, **p<0.01, ***p<0.001, ****p<0.0001.

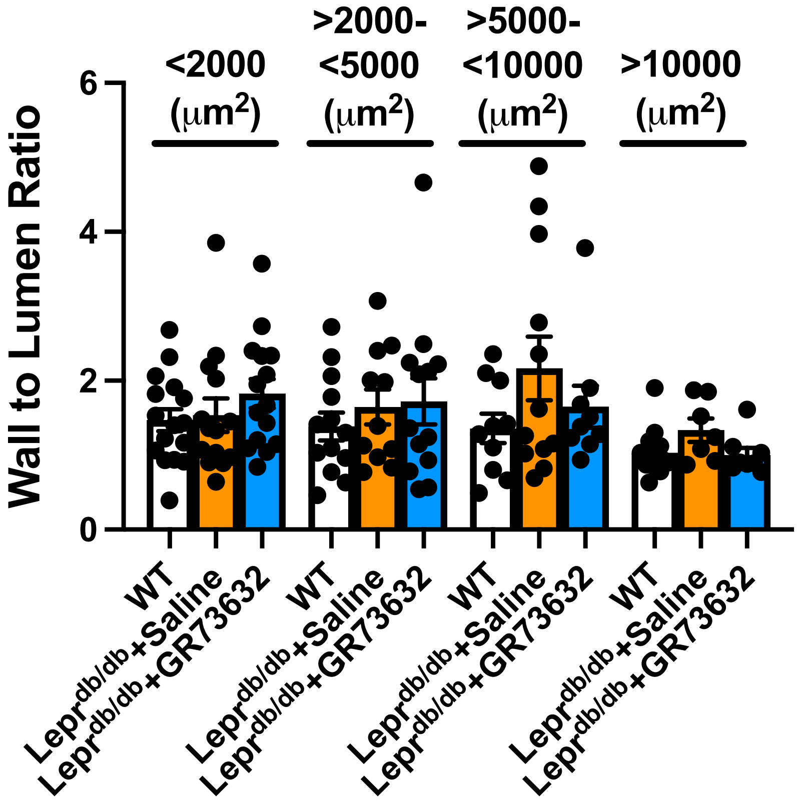

Supplemental Figure 4. Wall to lumen ratio for left ventricular intracardiac vessels in WT, Lepr$^{db/db}$+Saline and Lepr$^{db/db}$+GR73632 mice. All values are mean ± SD, n=10-12/group.

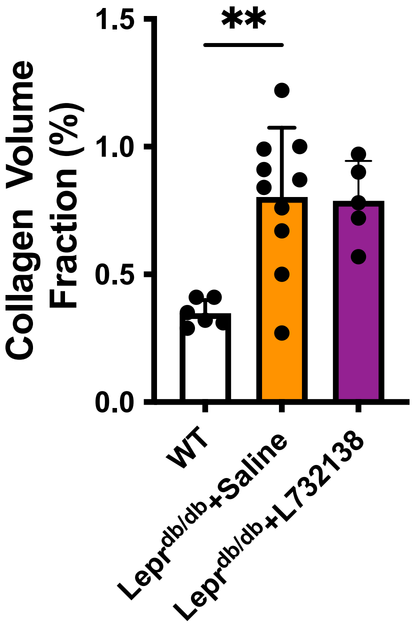

Supplemental Figure 5. The NK-1R antagonist, L732138 did not reduce cardiac fibrosis in Lepr$^{db/db}$ mice. Collagen volume fraction of WT (n=5), Lepr$^{db/db}$+Saline (n=10), Lepr$^{db/db}$ +L732138 (n=5). All values are mean ± SD, **p<0.01.

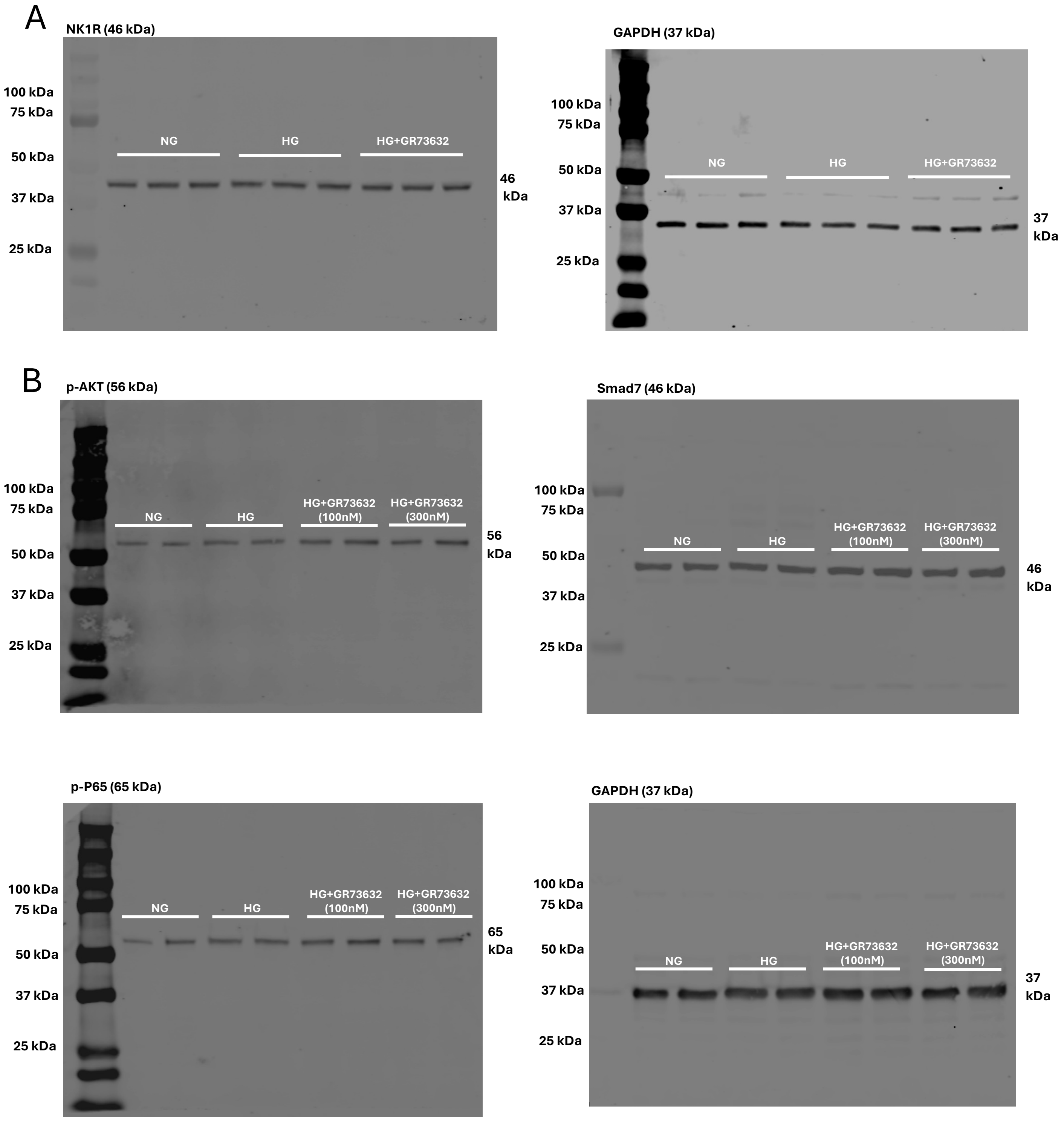

Supplemental Figure 6. Uncropped images of western blot membranes probed for (A) NK-1R with GAPDH; (B) p-AKT, Smad7, p-P65 with GAPDH.

Supplementary Table 1: Biometrics for 12 weeks old and 16 weeks old WT and Lepr$^{db/db}$ mice

	WT 12wks (mean ± s.d)	Lepr$^{db/db}$ 12wks (mean ± s.d)	WT 16wks (mean ± s.d)	Lepr$^{db/db}$ 16wks (mean ± s.d)
Body weight (gram)	27 ± 1.40	48.2 ± 5.58****	30.53 ± 1.92	44.8 ± 7.43$^{\#\#}$
Left ventricle index†	3.40 ± 0.37	3.36 ± 0.25	3.83 ± 0.47	3.36 ± 0.39$^{\#}$
Right ventricle index†	1 ± 0.12	1.12 ± 0.25	1.17 ± 0.19	0.96 ± 0.15$^{\#\#}$
Lung index†	5.12 ± 0.18	4.75 ± 0.21	5.73 ± 0.6	4.65 ± 0.57$^{\#\#\#\#}$
Left kidney index†	5.96 ± 0.65	7.27 ± 0.69	6.84 ± 1.02	6.71 ± 1.15
Right kidney index†	6.59 ± 0.60	8.03 ± 0.78*	7.31 ± 1.08	7.01 ± 1.10

†normalized to tibia length (mg/mm). *p<0.05, ****p<0.0001 versus WT 12wks. $^{\#}$p<0.05, $^{\#\#}$p<0.01, $^{\#\#\#\#}$p<0.0011 versus WT 16wks. n=8-23/group.

Supplementary Table 2: Proteins related to complement and coagulation cascades identified by KEGG analysis pathway of Lepr^{dbdb}+Saline vs WT LV

Entrez Gene ID	UniProt ID	Name	Symbol	Log2FC (Lepr$^{db/db}$ vs WT)
109828	D3YXF5	Complement component 7	C7	-1.23
110135	Q8K0E8	Fibrinogen beta chain	Fgb	-1.24
110382	Q8BH35	Complement component C8 beta chain	C8b	-1.93
11537	P03953	Complement factor D	Cfd	-2.09
11905	P32261	Antithrombin-III	Serpinc1	0.62
12258	P97290	Plasma protease C1 inhibitor	Serping1	-0.69
12260	P14106	Complement C1q subcomponent subunit B	C1qb	-0.39
12263	P21180	Complement C2	C2	-0.31
12266	P01027	Complement C3	C3	-0.96
12268	P01029	Complement C4-B	C4b	-0.61
12279	P06683	Complement component C9	C9	-1.58
12509	O55186	CD59A glycoprotein	Cd59a	-0.36
12628	P06909	Complement factor H	Cfh	-0.92
12759	Q06890	Clusterin	Clu	-0.53
13136	Q61475; Q61476	Complement decay-accelerating factor, GPI-anchored;Complement decay-accelerating factor transmembrane isoform	Cd55; Cd55b	-0.25
14058	O88947	Coagulation factor X	F10	-0.41
14161	E9PV24	Fibrinogen alpha chain	Fga	-1.30
16409	P05555	Integrin alpha-M	Itgam	-0.36
16644	O08677	Kininogen-1	Kng1	-0.30
17194	P39039	Mannose-binding protein A	Mbl1	-0.46
18815	P20918	Plasminogen	Plg	-0.77
18816	Q61247	Alpha-2-antiplasmin	Serpinf2	-0.92
20700	P07758	Alpha-1-antitrypsin 1-1	Serpina1a	-1.56
20703	Q00897	Alpha-1-antitrypsin 1-4	Serpina1d	-0.96
20704	Q00898	Alpha-1-antitrypsin 1-5	Serpina1e	-3.25
21824	P15306	Thrombomodulin	Thbd	-0.30
22370	P29788	Vitronectin	Vtn	-1.35
22371	Q8R2Z5	von Willebrand factor A domain-containing protein 1	Vwa1	-0.22
230558	Q8K182	Complement component C8 alpha chain	C8a	-2.03
56373	Q9JHH6	Carboxypeptidase B2	Cpb2	-0.44
74145	Q8BH61	Coagulation factor XIII A chain	F13a1	-0.49
99571	Q8VCM7	Fibrinogen gamma chain	Fgg	-1.19

Supplementary Table 3: Proteins related to autophagy identified by GO-biological processes of Lepr$^{db/db}$+GR73632 vs Lepr$^{db/db}$+Saline LV

Entrez Gene ID	UniProt ID	Name	Symbol	Log2FC (Lepr$^{db/db}$+ GR73632 vs Lepr$^{db/db}$+ Saline)
245860	Q68FE2	Autophagy-related protein 9A	Atg9a	0.24
52639	Q8R3E3	WD repeat domain phosphoinositide-interacting protein 1	Wipi1	-0.30
56486	Q9DCD6	Gamma-aminobutyric acid receptor-associated protein	Gabarap	0.29
66795	Q8R1P4	Ubiquitin-like-conjugating enzyme ATG10	Atg10	-0.04
108079	Q8BRK8	5'-AMP-activated protein kinase catalytic subunit alpha-2	Prkaa2	0.15
110157	Q99N57	RAF proto-oncogene serine/threonine-protein kinase	Raf1	0.14
11652	Q60823	RAC-beta serine/threonine-protein kinase	Akt2	0.13
12015	Q61337	Bcl2-associated agonist of cell death	Bad	0.15
18753	P28867	Protein kinase C delta type	Prkcd	0.15

Supplementary Table 4: Proteins related to membrane anchors identified by GO-cellular component of Lepr$^{db/db}$+GR73632 vs Lepr$^{d/bdb}$+Saline LV

Entrez Gene ID	UniProt ID	Name	Symbol	Log2FC (Lepr$^{db/db}$+ GR73632 vs Lepr$^{db/db}$+ Saline)
109979	Q8R2G4	Ecto-ADP-ribosyltransferase 3	Art3	-0.30
11647	P09242	Alkaline phosphatase, tissue-nonspecific isozyme	Alpl	-0.22
12509	O55186	CD59A glycoprotein	Cd59a	0.15
14723	O35930	Platelet glycoprotein Ib alpha chain	Gp1ba	-0.19
17067	P0CW02	Lymphocyte antigen 6C1	Ly6c1	-0.35
19122	P04925	Major prion protein	Prnp	-0.18
70574	Q80V42	Carboxypeptidase M	Cpm	0.16

Supplementary Table 5: Proteins related to responses to external stimuli identified by GO-biological processes of Lepr$^{db/db}$+GR73632 vs Lepr$^{db/db}$+Saline LV

Entrez Gene ID	UniProt ID	Name	Symbol	Log2FC (Lepr$^{db/db}$+ GR73632 vs Lepr$^{db/db}$+ Saline)
108079	Q8BRK8	5'-AMP-activated protein kinase catalytic subunit alpha-2	Prkaa2	0.15
110948	Q920N2	Biotin--protein ligase	Hlcs	-0.19
11364	P45952	Medium-chain specific acyl-CoA dehydrogenase, mitochondrial	Acadm	-0.20
18432	Q7TPV4	Myb-binding protein 1A	Mybbp1a	0.10
20656	P09671	Superoxide dismutase [Mn], mitochondrial	Sod2	-0.22
245860	Q68FE2	Autophagy-related protein 9A	Atg9a	0.24
52639	Q8R3E3	WD repeat domain phosphoinositide-interacting protein 1	Wipi1	-0.30
544791	B1AR69	Myosin, heavy polypeptide 13, skeletal muscle	Myh13	0.49
56367	Q78YZ6	Short coiled-coil protein	Scoc	0.32
66734	Q91VR7	Microtubule-associated protein 1 light chain 3 alpha	Map1lc3a	0.58

Supplementary Table 6: Chemical structure of SP, SP [7-11] and GR73632

	Chemical Structure
SP	Arg-Pro-Lys-Pro-Gln-Gln-Phe-Phe-Gly-Leu-Met-NH_2
SP [7-11]	Phe-Phe-Gly-Leu-Met-NH_2
GR73632	H_2N-(CH_2)4-CO-Phe-Phe-Pro-(Me)-Leu-Met-NH_2
